# Immune Cell Abundance and T-cell Receptor Landscapes Suggest New Patient Stratification Strategies in Head and Neck Squamous Cell Carcinoma

**DOI:** 10.1158/2767-9764.CRC-23-0155

**Published:** 2023-10-20

**Authors:** Maria Secrier, Lara McGrath, Felicia Ng, Sakshi Gulati, Amelia Raymond, Barrett R. B. Nuttall, Julie Berthe, Emma V. Jones, Ben S. Sidders, Jérôme Galon, J. Carl Barrett, Helen K. Angell

**Affiliations:** 1 Translational Medicine, Oncology R&D, AstraZeneca, Cambridge, United Kingdom.; 2 UCL Genetics Institute, Department of Genetics, Evolution and Environment, University College London, London, United Kingdom.; 3 Translational Medicine, Oncology R&D, AstraZeneca, Boston, Massachusetts.; 4 Oncology Data Science, Oncology R&D, AstraZeneca, Cambridge, United Kingdom.; 5 INSERM, Laboratory of Integrative Cancer Immunology, Paris, France.; 6 Sorbonne Université, Université Paris Cité, Centre de Recherche des Cordeliers, Paris, France.; 7 Equipe Labellisée Ligue Contre le Cancer, Paris, France.

## Abstract

Head and neck squamous cell carcinoma (HNSCC) is a molecularly and spatially heterogeneous disease frequently characterized by impairment of immunosurveillance mechanisms. Despite recent success with immunotherapy treatment, disease progression still occurs quickly after treatment in the majority of cases, suggesting the need to improve patient selection strategies. In the quest for biomarkers that may help inform response to checkpoint blockade, we characterized the tumor microenvironment (TME) of 162 HNSCC primary tumors of diverse etiologic and spatial origin, through gene expression and IHC profiling of relevant immune proteins, T-cell receptor (TCR) repertoire analysis, and whole-exome sequencing. We identified five HNSCC TME categories based on immune/stromal composition: (i) cytotoxic, (ii) plasma cell rich, (iii) dendritic cell rich, (iv) macrophage rich, and (v) immune-excluded. Remarkably, the cytotoxic and plasma cell rich subgroups exhibited a phenotype similar to tertiary lymphoid structures (TLS), which have been previously linked to immunotherapy response. We also found an increased richness of the TCR repertoire in these two subgroups and in never smokers. Mutational patterns evidencing APOBEC activity were enriched in the plasma cell high subgroup. Furthermore, specific signal propagation patterns within the Ras/ERK and PI3K/AKT pathways associated with distinct immune phenotypes. While traditionally CD8/CD3 T-cell infiltration and immune checkpoint expression (e.g., PD-L1) have been used in the patient selection process for checkpoint blockade treatment, we suggest that additional biomarkers, such as TCR productive clonality, smoking history, and TLS index, may have the ability to pull out potential responders to benefit from immunotherapeutic agents.

**Significance:**

Here we present our findings on the genomic and immune landscape of primary disease in a cohort of 162 patients with HNSCC, benefitting from detailed molecular and clinical characterization. By employing whole-exome sequencing and gene expression analysis of relevant immune markers, TCR profiling, and staining of relevant proteins involved in immune response, we highlight how distinct etiologies, cell intrinsic, and environmental factors combine to shape the landscape of HNSCC primary disease.

## Introduction

Despite recent advances in treatment options, head and neck squamous cell carcinoma (HNSCC) remains a multifaceted disease with variable outcome and a 10-year survival rate as low as 51%, depending on the subtype (1). Part of the difficulty in treating this cancer is its diversity in terms of site of origin along the oral and nasal cavities, pharynx and larynx, or associated etiology [human papillomavirus (HPV) positive/negative] (2). While HPV-positive patients show better survival outcomes (3), progress in understanding the causes for this has been hampered by the highly unbalanced datasets from this disease, with HPV-negative patients dominating the disease landscape (4). Both HPV infection and the site of cancer origin are likely to impact the tumor's interactions with its environment, stimulating or suppressing immune response and the ability of other cell populations to infiltrate the tumor (5). In fact, immune suppression seems to be a prevalent mechanism of neoplastic expansion in this cancer (6), which explains some of the recent successes in advanced-stage HNSCC with multiple checkpoint blockade agents such as pembrolizumab (7) or nivolumab (8). Alongside these factors, various other intrinsic cancer cell stimuli, as well as environmental risk factors, influence immune responses in this disease. For instance, tobacco smoking has been associated with lower T-cell cytotoxicity and exhaustion in HNSCC (9). Understanding how various risk factors and genomic events act together to shape tumor immunity at the site of primary disease in HNSCC and subsequent development of T-cell exhaustion phenotypes (10) may lead to a more accurate identification of patients who have the potential to develop long-lasting antitumor immunity.

There is abundant evidence in the literature that the genomic make-up of the tumor can impact immune responses, with a higher tumor mutational burden reflecting a possible enhanced neoantigen presentation, which has already been successfully exploited as a biomarker for checkpoint blockade in a variety of cancers (11–13). Furthermore, advances in the area of T-cell receptor (TCR) profiling (14–16) and recent efforts in the characterization of tertiary lymphoid structures (TLS) in multiple cancers (17) have brought to attention the myriad of tumor evasion and recognition mechanisms within the tumor microenvironment (TME) that could be exploited to enhance immune responses. Expanding the understanding of these complex TME interactions in head and neck cancer will enable us to derive genetic, expression, and/or clinical biomarkers that can inform more effectively on the state of activity of the TME and on the potential for enhancing immunity. Ultimately, this knowledge should help patient selection strategies and potential opportunities for deployment of novel testing approaches that can be employed earlier in the course of the disease.

Here we present our findings on the genomic and immune landscape of primary disease in a cohort of 162 patients with squamous cell carcinoma of the head and neck, benefitting from detailed molecular and clinical characterization. We have aimed to maximize the diversity of phenotypes observed by ensuring a broad and comprehensive distribution of primary tumor samples originating from several sites along the oro-nasal cavities, pharynx and larynx, as well as balancing the proportion of HPV-positive and -negative cases (Supplementary Table S1). By employing whole-exome sequencing and matched gene expression analysis of relevant immune markers, as well as TCR profiling and staining of relevant proteins involved in immune response, we highlight how distinct etiologies, cell intrinsic, and environmental factors combine to shape the landscape of primary disease in HNSCC.

## Materials and Methods

### Acquisition of Samples

All human tissues were obtained with full written informed consent and transferred to AstraZeneca. AstraZeneca has a governance framework and processes in place to ensure that commercial sources have appropriate patient consent and ethical approval in accordance with the principles outlined in the Declaration of Helsinki, in place for collection of the samples for research purposes including use by for-profit companies. The AstraZeneca Biobank in the United King is licensed by the Human Tissue Authority (License No. 12109) and has National Research Ethics Service Committee approval as a Research Tissue Bank (REC No. 17/NW/0207) which covers the use of the samples for this project.

### Immune Gene Expression Profiling

A total of 162 primary tumor sample sections were submitted for NanoString sequencing using the PanCancer Immune Profiling Panel (800 genes). The resulting data were processed using the nSolver Analysis Software (https://www.nanostring.com/products/analysis-software/nsolver) from NanoString.

### IHC

CD8^+^/CD3^+^ T-cell and regulatory T cell infiltration were evaluated using IHC by staining for CD8, CD3, FOXP3, and GITR, respectively. The abundance of these cells at the center of the tumor and at the invasive margin were quantified separately. We also stained for PD-L1 (SP263) expression.

### TCR Repertoire

The TCRβ repertoire was assessed in *n* = 68 samples with sufficient material available from the cohort through a targeted DNA sequencing assay (immunoSEQ, Adaptive Biotechnologies; refs. 18, 19). The sample selection was agnostic to clinical characteristics of the respective patients (smoking status, age, etc.). Three samples were subsequently removed from the analysis due to low DNA input and low T-cell numbers (<100 CDR3 sequences recovered). Productive rearrangements were derived from in-frame CDR3 sequences, sequences lacking frameshifts or stop codons. Only productive rearrangements were used for calculations of richness, clonality, and clonal frequency. Richness was calculated by the total number of unique CDR3 rearrangements. Clonality (reciprocal of Shannon entropy) is defined on a scale from 0 to 1 (low to high), and describes the sample richness and the evenness in distribution of the clonal abundance.

### Whole-exome Sequencing and Variant Calling

DNA extraction of the formalin-fixed paraffin embedded (FFPE) sections with the Omega Bio-tek Mag-Bind FFPE DNA Kit was performed on a KingFisher Flex instrument. Extracted DNA was quantitated using the KAPA Human Genomic DNA Quantification and QC Kit, and stored at −20°C. Whole-genome libraries were constructed using the KAPA HyperPlus Kit using a Beckman Coulter Biomek FxP liquid handler. Unique dual-indexed adapters containing a 6 bp unique molecular identifier (UMI) sequence, sourced from Integrated DNA Technologies (IDT), were ligated to the fragmented DNA. Libraries were quantitated using both the Agilent TapeStation D1000 assay and the KAPA Library Quantification Kit (qPCR). Hybridization capture was performed to enrich for the exonic, coding regions of the human genome. Before capture, whole-genome libraries were multiplexed, six samples per pool, with a total input of 2,000 ng per capture. The hybridization capture was performed manually using the IDT xGen Exome Research Panel v1.0 and the Roche NimbleGen Hybridization and Wash Kit. Whole-exome libraries were quantitated using both the Agilent TapeStation D1000 assay and the KAPA Library Quantification Kit (qPCR). Whole-exome libraries were sequenced using 2 × 150 chemistry on an Illumina HiSeq 4000 instrument.

FFPE samples (*n* = 82) were whole-exome sequenced in-house. Data were analyzed using pipeline software bcbio-nextgen v1.1.1 (https://doi.org/10.5281/zenodo.5781867). Reads were aligned to the hg38 reference using bwa v0.7.15, a quality control (QC) report was generated using multiqc, and sequencing duplicates for each UMI were collapsed into a single consensus read using fgbio. No samples were subsequently excluded from the analysis due to low quality (as judged using sequencing depth of coverage <50x and on-target reads <50%). Median coverage for the 82 samples was 91x. Variant calling was performed using VarDict v1.5.4 (20), down to a variant allele frequency (VAF) of 1% (before filtering and curation) and variant effects annotated by snpEff v4.3.1t. Filtering of non-cancer variants (i.e., common polymorphisms) was performed as per VarDict best practice. In addition, variants were removed if it satisfies any one of the following criteria: (i) VAF >95%, (ii) VAF <5%, (iii) <4 alternative reads supporting the variant, (iv) variant depth <50. Potential artefacts induced by the fixing procedure were corrected using the DKFZ bias filter (https://github.com/DKFZ-ODCF/DKFZBiasFilter), such that single-nucleotide variants (SNV) appearing to be biased in terms of variant read support were removed.

### Tumor Subclonality Assessment

Because of the fact that identification of tumor subclones in individual bulk sequenced samples is prone to inaccuracy, we aimed to obtain a simple estimation of evidence for the existence of subclonality in a tumor rather than an exact quantification of the number of subclones and their mutational make-up. The VAF distribution is informative for this purpose, with peaks closer to 1 indicating the clonal population and peaks deviating from that corresponding to subclones. We sought to identify any multimodal distribution in the data, which would likely be indicative of two or more separate subpopulations rather than the existence of a long tail of neutral mutations. To assess deviations from unimodality, we used Hartigan dip test (21) as implemented in the diptest R package.

### Mutational Signatures

Mutational signatures were inferred using deconstructSigs (22), based on the single base substitution (SBS) patterns reported in the COSMIC database (v3—May 2019; ref. 23).

### Tumor Immunity Profiling

The composition of the TME was assessed on the basis of expression profiles using ConsensusTME (24) and HNSCC cell type–specific signatures. RNA-sequencing transcripts per million (TPM) values were obtained for 520 HNSCC primary tumors from The Cancer Genome Atlas (TCGA) for validation using the TCGA2STAT R package (25). Immune infiltrates were estimated using the ConsensusTME package, and immunity groups were defined using the ConsensusClusterPlus R package with the same parameters as in the discovery cohort.

### Modeling Pathway Signaling Impact on Tumor Immunity

Multinomial logistic regression was employed to associate expression of genes in the Ras/ERK and PI3K/AKT pathways with observed tumor immunity phenotypes. The expression of 29 genes in these pathways had been measured on the NanoString platform. Initially, the expression of these genes was employed to model four immunity outcomes [immune-excluded, weak, exhausted, plasma cell (PC) high] using multinomial regression. In this initial modeling approach, it was observed that the model underperformed in distinguishing the intermediate phenotypes “weak” and “exhausted” (overall accuracy 63%). This suggested that there may be other factors beyond kinase signaling involved in determining the levels of infiltration and exhaustion, and therefore that this signaling may only help distinguish high and low immune activity. When modeling these two distinct immune states based on the expression of the top genes that show significant difference in expression between the two states, the accuracy jumped to 81%.

### TLS Signatures

The TLS signatures were manually curated from the literature, with focus on pan-cancer rather than tissue-specific markers (26–28, 29). The employed signatures are documented in Supplementary Table S2. A TLS score per sample was defined as the mean expression across the TLS gene signature markers and the TLS chemokine markers, respectively. The TLS scores were validated in the corresponding immunity groups identified in 520 HNSCC samples from TCGA.

### Statistical Analysis

Groups were compared using the Wilcoxon rank-sum test and Kruskal–Wallis test, where appropriate. Enrichment analyses were conducted using the Fisher exact test. Multiple testing corrections were performed on the basis of the Benjamini–Hochberg method. Survival analysis was performed through Cox proportional hazards univariate and multivariate modeling.

### Data Availability

Data underlying the findings described in this article may be obtained in accordance with AstraZeneca's data sharing policy described at https://astrazenecagrouptrials.pharmacm.com/ST/Submission/Disclosure. Requests to access the data described in the current article can be submitted through https://vivli.org/members/enquiries-about-studies-not-listed-on-the-vivli-platform/.

## Results

### Cancer Etiology Shapes Immune Phenotypes and Survival Outcomes in HNSCC

Immune and stromal cell abundance in the TME is an important determinant of immune response capacity in cancer and has been linked with prognosis in multiple cancer tissues (30, 31). A further indicator of the ability of the body to mount an immune response is the TCR repertoire, with increased productive clonality within the TME suggesting accumulation of tumor antigen–specific T cells. Recent studies demonstrate that the intratumoral TCR repertoire is prognostic of response to anti-PD-1 and anti-CTLA-4 therapies (14). We aimed to characterize the immune composition of the TME and the TCR capacity in conjunction in the HNSCC cohort. Using expression data for a panel of 800 immune-related genes, we evaluated inflammation programs in 162 primary tumor samples collected within the cohort. By employing immune cell–specific signatures and TME deconvolution methods on bulk expression data (see Materials and Methods), we inferred the abundance and activity of multiple cell populations in the TME of these tumors. Subsequently, we examined the pretreatment intratumoral T-cell β repertoires in a subset of 68 HNSCC patient samples together with clinical data and other biomarkers, such as expression of inhibitory checkpoints.

**FIGURE 1:**
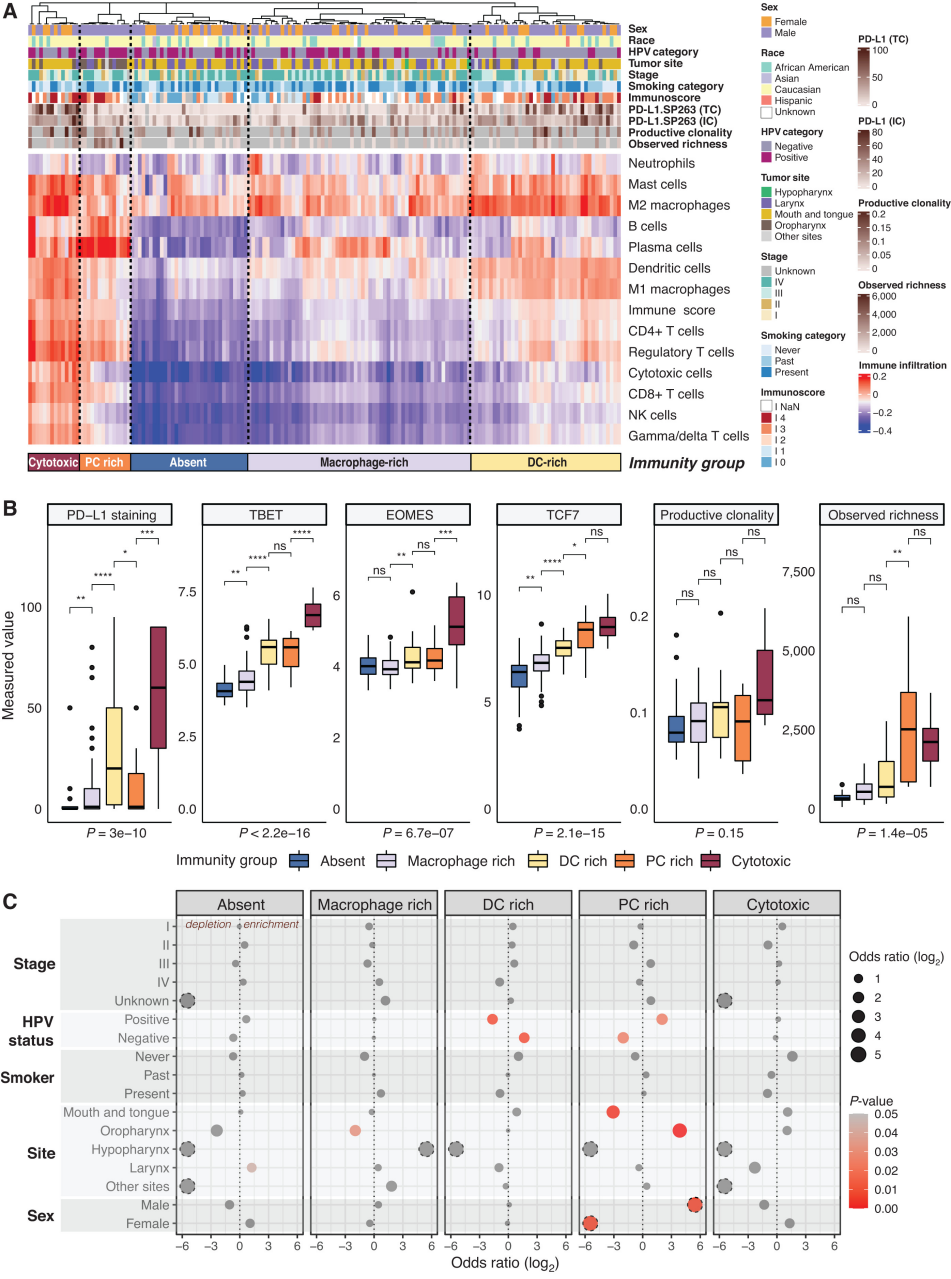
Emerging TME phenotypes and their characteristics in the HNSCC cohort. **A,** The heat map displays the immune cell enrichment/depletion in bulk tumor samples from the cohort, as inferred from the expression of cell type–specific markers using ConsensusTME and ssGSEA (single-sample gene set enrichment analysis). Every column corresponds to a sample and every row depicts an immune subpopulation. Samples are clustered on the basis of the predicted enrichment/depletion of immune cell populations. Relevant clinical features, Immunoscore (CD3 and CD8 quantification), PD-L1 staining by IHC (TC – tumor cell and IC – immune cell positivity) and TCR repertoire characteristics (productive clonality, observed richness) are annotated. Five subgroups with distinct immunity emerge: highly cytotoxic and exhausted, PC rich, DC rich, macrophage rich, and immune-excluded. Key clinical characteristics are also annotated. **B,** Markers of immune exhaustion and TCR repertoire differ significantly among the immune subgroups defined in A. Higher TCR clonality and exhaustion is generally observed in the highly cytotoxic group (exhausted, PC high). The *y* axis depicts PD-L1 staining measurements by IHC; TBET, EOMES, and TCF7 expression from NanoString; and computed TCR productive clonality and observed richness, respectively. Kruskal–Wallis test *P* values comparing the five groups are displayed underneath the plots. ****, *P* < 0.0001; ***, *P* < 0.001; **, *P* < 0.01; *, *P* < 0.05; ns, nonsignificant (*P* > 0.05). **C,** Enriched characteristics in the five immunity subgroups. Red circles denote significant enrichments from a Fisher exact test. Dotted circles represent infinite ORs.

The microenvironment of pretreatment HNSCC primary tumors displayed a spectrum of immune contexture that could be broadly grouped into five subclasses, evidencing high T-cell cytotoxicity and exhaustion (9%), enrichment of PC (9%), dendritic cells (DC, 25%), and macrophages (37%), as well as an immune-excluded subgroup (20%; ref. Fig. 1A; Supplementary Fig. S1). These five subclasses emerged as a result of clustering the samples based on the overall immune and stromal content, and displayed strikingly distinct levels of immune exhaustion, as evidenced by PD-L1 protein expression and levels of classical exhaustion markers such as TBET, EOMES, and TCF7 (refs. 10, 32; Fig. 1B). Furthermore, the productive clonality and observed richness of the TCR repertoire, calculated as the total number of unique CDR3 rearrangements, tended to increase with increasing immune potential of the group (Fig. 1B). The TCR richness peaked in the groups enriched in PCs and cytotoxic cells, suggesting both of these groups might have a higher potential for a sustained immune response. Overall within the cohort, we observed strong associations between immune cell populations generally associated with cytotoxic microenvironments such as CD8^+^/CD4^+^ T cells, natural killer (NK) cells, regulatory T cells (Supplementary Fig. S2). There was also a slight inverse correlation between PCs and M2 macrophages.

A similar immune landscape was confirmed in an independent cohort of 520 HNSCCs from TCGA (Supplementary Fig. S3), with the same immunity groups emerging in comparable proportions: immune-excluded (a total of 24% in TCGA vs. 30% in the discovery cohort), macrophage rich (43% vs. 37%), DC enriched (20% in TCGA and 25% in the discovery cohort), PC enriched (11% in TCGA vs. 12% in the discovery cohort), and cytotoxic (2% and 9%, respectively). The groups defined in TCGA also showed significant differences in PD-L1 expression, with highest levels in the cytotoxic, PC and DC rich subgroups, similar to what was observed in our study cohort (Supplementary Fig. S4).

Within the proposed immunity groups in the discovery cohort, we observed prominent differences in terms of clinical and tumor characteristics (Fig. 1C). Specifically, the PC high group showed an enrichment of HPV-positive cancers of oropharynx origin and was exclusively observed in male patients. Conversely, there was an enrichment of HPV-negative patients in the DC rich group. There was no difference in immunity group distribution by stage or smoking status, and the two most common ethnicities in this cohort, Caucasian and African American, were represented in every immunity group These results suggest that cancer etiology influences tumor immunity in HNSCC, with the HPV status and site of origin being the strongest determinants of immune architecture (Supplementary Fig. S5 and S6). Specifically, samples of laryngeal origin showed the lowest cytotoxicity (lowest CD8^+^/CD4^+^ T-cell and NK-cell infiltration) and high prevalence of tumor-promoting M2 macrophages and neutrophils, while the oropharynx samples exhibited opposite trends.

Concordantly, significant differences in overall survival were found between the five subgroups after correcting for potential confounders such as cancer stage, sex, age, or smoking habits in a multivariate Cox proportional hazards model (Supplementary Table S3; Supplementary Fig. S7). Within this model, the smoking status and tumor recurrence also had a significant effect on survival. As expected, the immune-excluded group has the worst prognosis, with the high cytotoxicity and macrophage enriched groups showing significantly better overall survival. When analyzed by these five subgroups, differences in survival outcomes were similarly observed in TCGA cohort (Supplementary Fig. S8).

### APOBEC Activity Drives HNSCC Mutagenesis and PC Enriched Phenotypes

To understand whether any genomic drivers may explain the distinct TME phenotypes we observe in HNSCC, we performed whole-exome sequencing of available samples in the cohort and sought to identify mutations that may be associated with higher or lower immune capacity.

**FIGURE 2:**
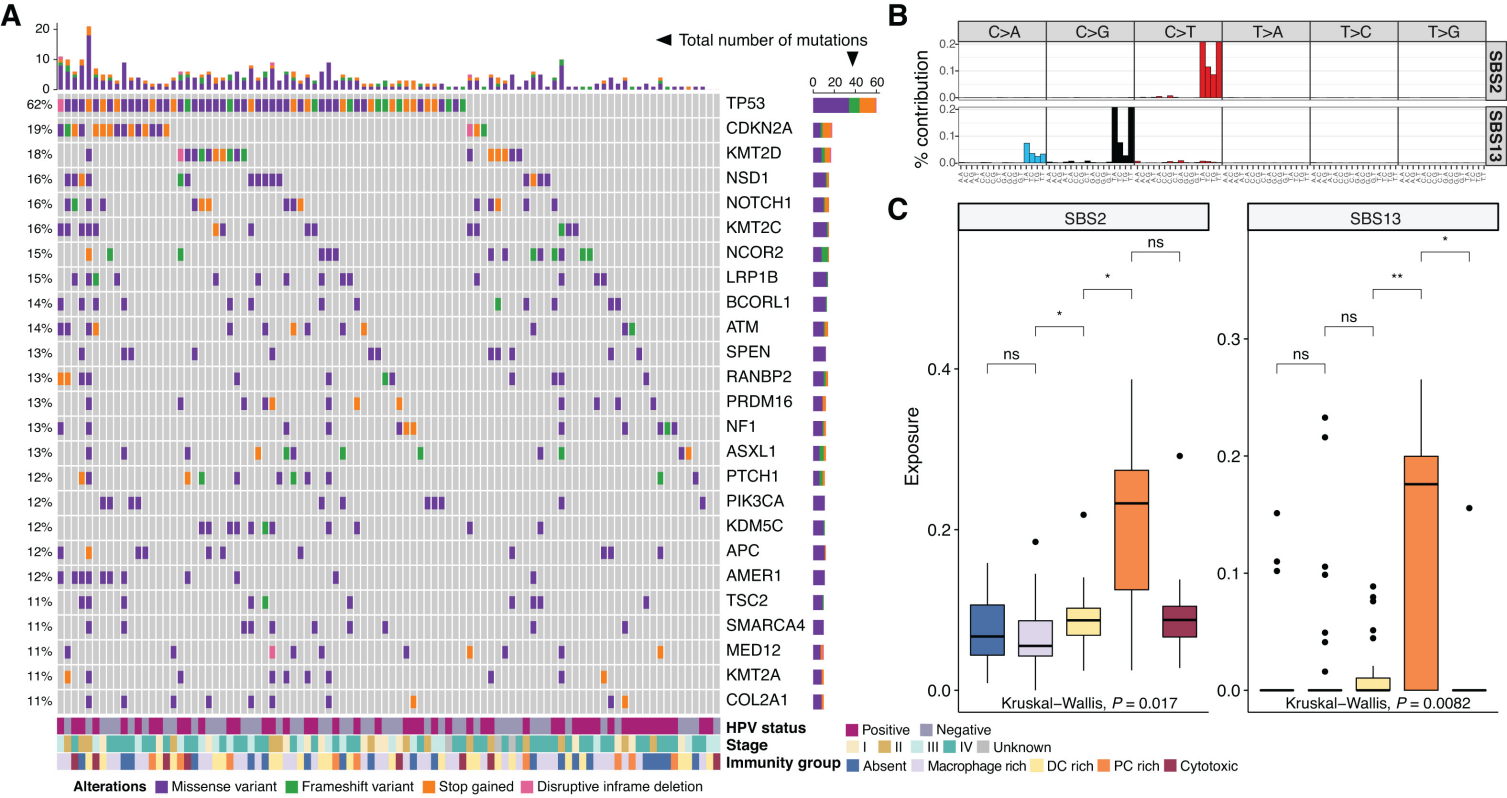
Genomic processes in the HNSCC cohort. **A,** Driver genes recurrently altered (by SNV/indel) in more than 10% of the samples are shown, along with key clinical (HPV status, stage) and molecular characteristics (immunity subgroup). The bar plots display the total number of mutations per gene (rows) and sample (columns), respectively, across the cohort. **B,** The pattern of mutational probabilities for the 96 base substitution contexts characterizing the mutational signatures SBS2 and SBS13, showing distinct prevalence across immunity subgroups, is displayed. **C,** The SBS signatures associated with mutational processes 2 and 13 from the COSMIC database, linked with AID/APOBEC activity, show differing contributions of mutations by immunity subgroup, with an increase in the PC rich subgroup (boxplot subgroup colors as in A). **, *P* < 0.01; *, *P* < 0.05; ns, nonsignificant (*P* > 0.05).

Overall, from a genomic perspective the cohort displayed, the general level of heterogeneity expected in this type of cancer. The total mutational burden varied between 83 and11,780 mutations per tumor, with a median of 353 mutations and median absolute deviation of 229.8. The mean mutation rate resembled that reported in the literature for HNSCC [6.2 mutations/Mb vs. ∼7 mutations/Mb (33)]. There was no significant difference in mutational burden across the immunity subgroups (Supplementary Fig. S9). While no association between tumor mutational load and immune contexture (as measured by TCR productive clonality or PD-L1 staining) was observed, there was a moderate inverse correlation with the observed richness of the TCR repertoire (*P* = 0.015; Supplementary Fig. S10). In terms of cancer drivers, TP53 was the most frequently altered gene as expected (62% of the cohort), followed by p16 (CDKN2A, 19%), KMT2D, NSD1 and NOTCH 1 (18% each; Fig. 2A).

There was a 4.7-fold enrichment of TP53 wild-type samples in the HPV-positive group (Fisher exact test *P* = 0.005), confirming reports in the literature associating HPV infection with a functional TP53 protein background (2, 4). This was the only gene whose mutations status varied significantly according to HPV infection status. TP53 mutations were depleted in the PC high group compared with the rest (Fisher exact test *P* = 0.002), congruent with the HPV positivity enrichment highlighted in the previous section. None of the other driver genes were significantly enriched by HPV status or immunity group after multiple testing correction.

Examination of mutation allele frequency distributions suggested frequent subclonality in these tumors: 76 out of 95 tumors, that is, 80% of the cohort, displayed a pattern of mutations consistent with the existence of at least one subclone (Hartigan dip test for unimodality adjusted *P* values <0.05, see Materials and Methods). This may account for some of the heterogeneity observed in terms of genetic drivers. Interestingly, tumors with evidence of subclonality had a significantly higher TCR productive clonality (Supplementary Fig. S11). No significant associations were observed with other exhaustion and TCR repertoire markers (Wilcoxon rank-sum test *P* > 0.05) or with immunity subgroups (Fisher exact test *P* > 0.05).

To gain insight into potential causative risk factors of HNSCC in our cohort and the deregulated pathways involved in the process, we subsequently investigated the mutational processes that have shaped the genomes of these patients. Recent efforts (33, 34) have delineated evidence from various mutational processes contributing to cancer development in the form of genome-wide trinucleotide substitution patterns, which associate with various types of DNA damage and are indicative of environmental and intrinsic neoplastic risk factors (33, 35). As such, ageing and APOBEC enzymatic activity–related signatures have previously been reported in HNSCC whole-exome sequenced samples (36, 37), with rarer contributions from smoking or oxidative stress–related signatures SBS4 and SBS18 highlighted in the most recent pan-cancer analysis of whole genomes (PCAWG) study (34). We used deconstructSigs (22) to infer mutational signatures in our cohort from whole-exome sequencing data and identified prevalent signals associated with DNA-editing activity of the AID/APOBEC cytidine deaminase family (signatures SBS2 and SBS13 in COSMIC, Fig. 2B), with APOBEC3A believed to be the main catalyser of such mutations in human cancers and a potentially more minor role from APOBEC3B (38, 39, 40). Other encountered signatures were linked with ageing (SBS1/5), base excision repair (SBS30), mismatch repair deficiencies (SBS6, SBS44), and polymerase epsilon activity (SBS10b), among others (Supplementary Fig. S12). Notably, the APOBEC-associated signatures SBS2 and SBS13 showed the highest contribution in PC rich samples (Fig. 2C), and SBS2 was elevated in HPV-positive samples (Student one-tailed *t* test *P* = 0.02) confirming the previous report by Henderson and colleagues (36). APOBEC has been previously linked to immunotherapy response in lung cancer (41), suggesting this mechanism may be involved in the generation of immunity for cancers with high infiltrate of PCs.

### Ras/ERK and PI3K/AKT Signaling Modulate Immune Phenotypes

Multiple immune phenotypes are observable in the cohort, but the question remains to what extent the properties of the tumor itself may be determining these phenotypes. As few genomic drivers were found to be associated with distinct immune phenotypes, we asked whether signaling within the tumor or the broader TME may be shaping immune responses to some extent. To answer this question, we focused on the Ras/ERK and PI3K/AKT signaling pathways which have been previously implicated in HNSCC carcinogenesis (2). We observed that several of the genes in these pathways were mutated (Supplementary Fig. S13), suggesting that this may be one of the mechanisms construed to drive tumor development in this cohort. Interestingly, while there was no clear correlation with TCR productive clonality (Supplementary Fig. S14), many of these genes showed significant positive (PIK3CD, PIK3CG) or negative (IGF1R, MET) correlations with the observed richness of the TCR repertoire (Supplementary Fig. S15). We therefore hypothesized oncogenic signaling across these pathways may also have an impact on the TME.

**FIGURE 3:**
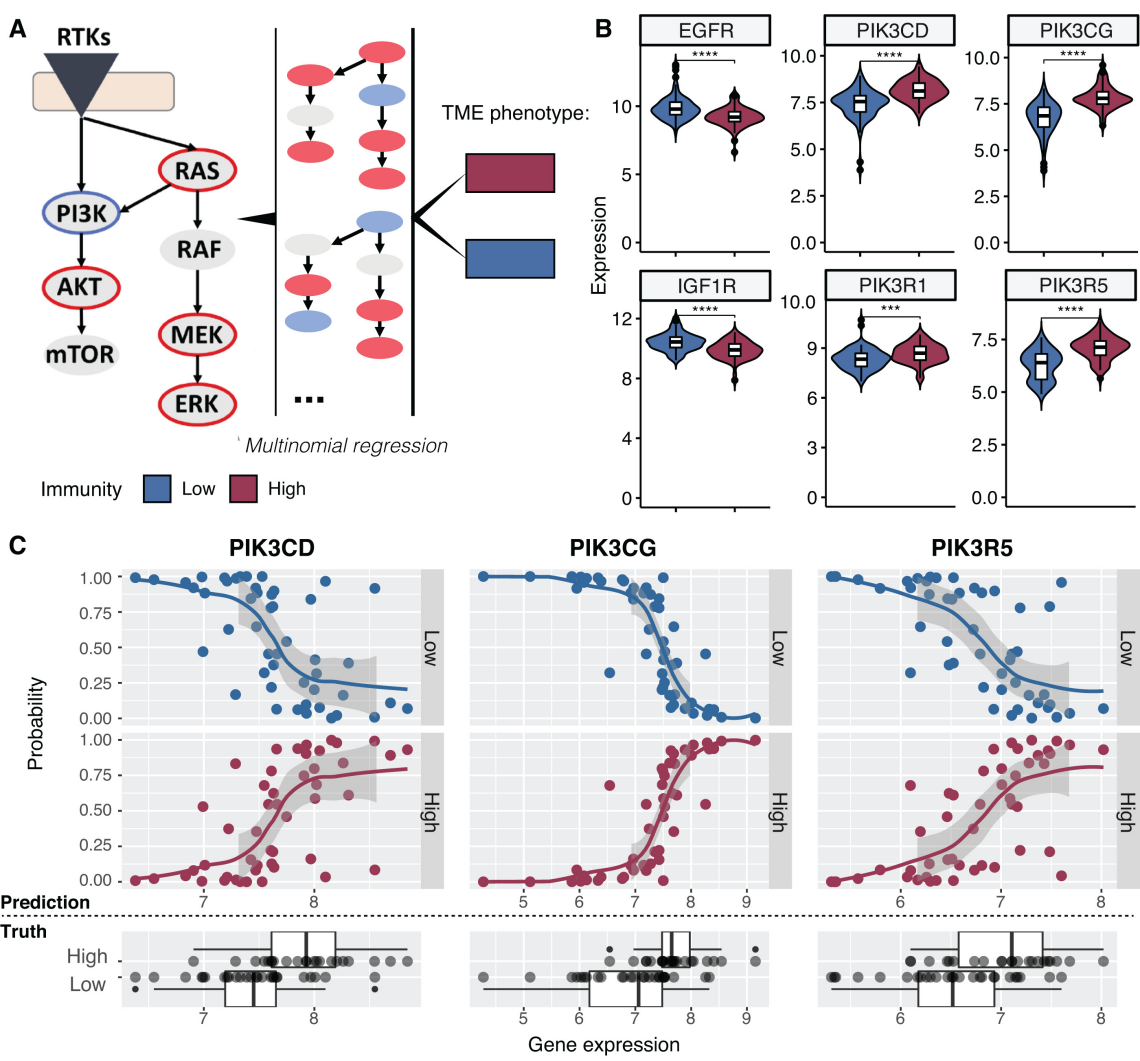
Modeling TME outcomes based on signaling in tyrosine kinase pathways. **A,** Multinomial logistic regression model that was applied to integrate expression signals across relevant pathways to predict the immunogenic/high immunity (cytotoxic, PC/DC rich) versus low immunity (immune-excluded/macrophage rich) phenotypes (as indicated by the color legend). **B,** Example genes in the PI3KA/AKT pathway that are significantly differently expressed among immunity subgroups. **C,** The genes with most significant trends included in the final model and their predictive power to distinguish between immunity subgroups. The top panel shows the predicted immunity state (low/high) depending on expression of individual genes in the model; the bottom panel shows the true distribution of expression values for the respective genes among the high and low immunity groups. The curves in the prediction model were fitted with a loess function. Extreme values of the expression distribution seem to most clearly define immune state.

Because the expression of several genes in the pathway had been measured in the panel, this allowed us to assess differences in cellular signaling among the distinct TME classes. We indeed observed significant differences, including elevated levels of PIK3CD, PIK3CG, and PIK3R5, and a decrease in EGFR, IGF1R, and MET in the group of tumors with higher immunity (Supplementary Fig. S16). The fact that a low EGFR expression tends to be associated to an immune “hot” environment suggests the possibility of a dual positive effect for EGFR inhibition in EGFR-activated HNSCCs, where tumor cell growth could be suppressed alongside an immune response enhancement. A similar trend was observed in TCGA (Supplementary Fig. S17). Subsequently, we used multinomial logistic regression to model high/low immunity based on combinations of Ras/ERK and PI3K/AKT signaling (Fig. 3A), and identified IGF1R, KRAS, MAPK1, PIK3CD, PIK3CG, and PIK3R5 combined expression as significant predictors of tumor immunity with an accuracy of 81% (Akaike information criterion [AIC] = 76.2; Supplementary Fig. S18). In particular, we observe that extreme values of the PIK3CD, PIK3CG, and PIK3R5 genes (either at the lower end or higher end of the expression spectrum) are highly effective in combination in determining the immune phenotype (Fig. 3B and C). These results suggest an interplay between PI3K pathway–driven cell growth and immunity that could potentially be exploited for therapy.

### TLSs and TCR Repertoire Delineate Immune “hot” Tumors

**FIGURE 4:**
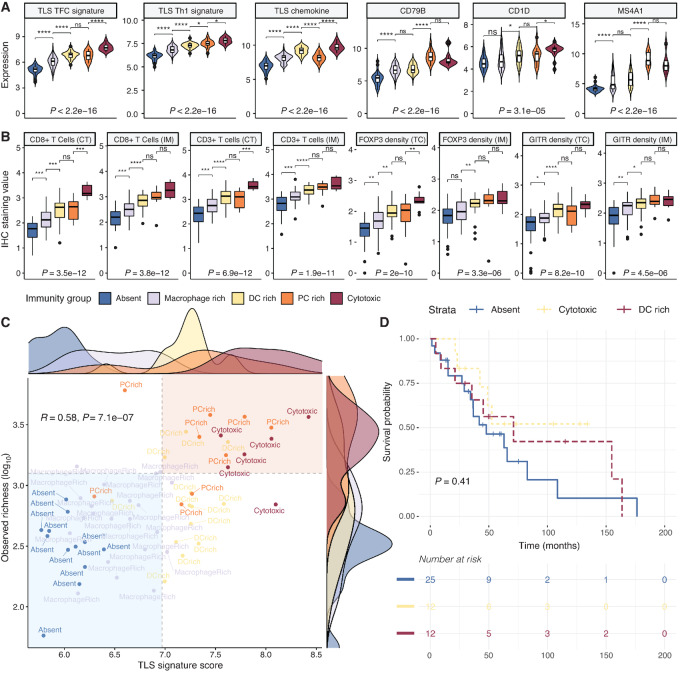
TLS signatures and TCR repertoire richness as markers of immune “hot” tumors with improved survival outcomes. **A,** Expression of TLS-specific signature genes and chemokine signaling appears elevated in the cytotoxic and PC high subgroups. Other individual markers associated with TLSs (28) display similar trends. ****, *P* < 0.0001; ***, *P* < 0.001; **, *P* < 0.01; *, *P* < 0.05; ns, nonsignificant (*P* > 0.05). **B,** Protein staining for CD8^+^/CD3^+^ T cells and regulatory T cell markers (FOXP3, GITR) by IHC shows increased levels in the cytotoxic groups. TC, staining at the tumor center; IM, staining at the immune margin. Boxplots compare staining levels between independent samples across the five groups: immune-excluded, *n* = 32; macrophage rich, *n* = 61; DC rich, *n* = 41; PC rich, *n* = 14; cytotoxic, *n* = 4. **C,** The TLS signature score is strongly correlated with the observed richness of the TCR repertoire. A grouping of tumor TME subtypes emerges from these two parameters. The density plots along the scatter plot depict the distribution of TLS signature scores and observed TCR richness, respectively, for every immune category. The upper right corner of the scatter plot, comprising all cytotoxic samples, corresponds to an immune “hot” state with high evidence of TLS and high richness of the TCR repertoire. The bottom left corner of the scatter plot is enriched in samples with excluded/weak immunity. **D,** Patients with an “immune-excluded” phenotype (as classified in B) present a trend for worse overall survival outcomes, while highly cytotoxic tumors confer a survival advantage.

The previous analyses have demonstrated distinctive genomic and immunity features in the different immunity groups that are worthy of further investigation. Recent literature has highlighted improved immunotherapy response in the presence of TLSs (26–28). In cancer, TLSs are highly organized lymphoid aggregates resembling lymph nodes that develop in settings of inflammatory conditions and act as sites of lymphoid recruitment and immune activation (17). Composed of multiple immune cell types such as T cells, B cells, mature DCs, as well as differentiated stromal cells and endothelial cells (17), TLSs have been described to be particularly rich in PCs (42–45) and are often found in association with CD8^+^ T cells (46–48) and regulatory T cells (49–51). We hypothesized that the strong prevalence of PCs in the PC high group might be associated with such structures. To test this, we manually curated TLS core gene and chemokine signatures from the literature (28, 29) that were generic rather than cancer tissue dependent and had been measured in our expression experiments (Supplementary Table S2). Subsequently, we assigned a TLS signature score for T follicular cells (TFC) and Th1 helper cells, as well as a TLS chemokine score to every tumor in the cohort based on the average expression of genes included in the respective signatures. We observed a progressive increase in TLS scores with increasing immunogenicity, peaking in the cytotoxic group (Fig. 4A). Several additional B-lineage markers previously shown to be associated with the presence of TLSs (28), such as CD79B, CD1D, and MS4A1 (CD20), were highest expressed in the cytotoxic and PC high groups (Fig. 4A).

We observed similar increases in IHC protein staining for CD8^+^ and CD3^+^ T cells (Immunoscore), as well as FOXP3/GITR levels in the DC rich, PC rich, and cytotoxic groups (Fig. 4B), reflecting an increased abundance of the corresponding immune cells (CD8^+^/CD4^+^ and regulatory T cells). The same trends were observed at expression level for the respective genes (Supplementary Fig. S19) and were maintained in tumors regardless of HPV status (Supplementary Fig. S20 and S21). This would suggest there exists a good potential for TLS formation in both the cytotoxic and PC high groups. Similar trends were observed in 520 HNSCC samples from TCGA, with the PC high group displaying the highest TLS signature score average (Supplementary Fig. S22). Interestingly, the TLS signature showed a significant correlation with PD-L1 status, the productive clonality and observed richness of the TCR repertoire (Fig. 4C; Supplementary Fig. S23). The large majority of cytotoxic samples had high TLS score and high observed TCR richness. Remarkably, the samples with low TLS score and low TCR richness were enriched in the immune-excluded group and showed a trend for worse overall survival, while cytotoxic tumors with high TCR richness and TLS expression linked with the best outcome (Fig. 4D). These features indicative of immune “hotter” phenotypes would be worth exploiting in a clinical context, as these patients may demonstrate improved responses to immunotherapy. However, due to different expression levels of these TLS core genes and chemokines being reported across TLS models (17), further validation of this TLS gene signature is warranted, to ensure its true association with defined TLS. More TLS features, such as TLS composition and maturation status, TLS location within the tumor as well as the inflammatory context, should also be taken into account using multiplexing technologies to better understand the relationship between TLSs, immune potential and clinical outcome.

### Cancer Risk Factors Contribute to Observed Differences in HNSCC Tumor Immunity

**FIGURE 5:**
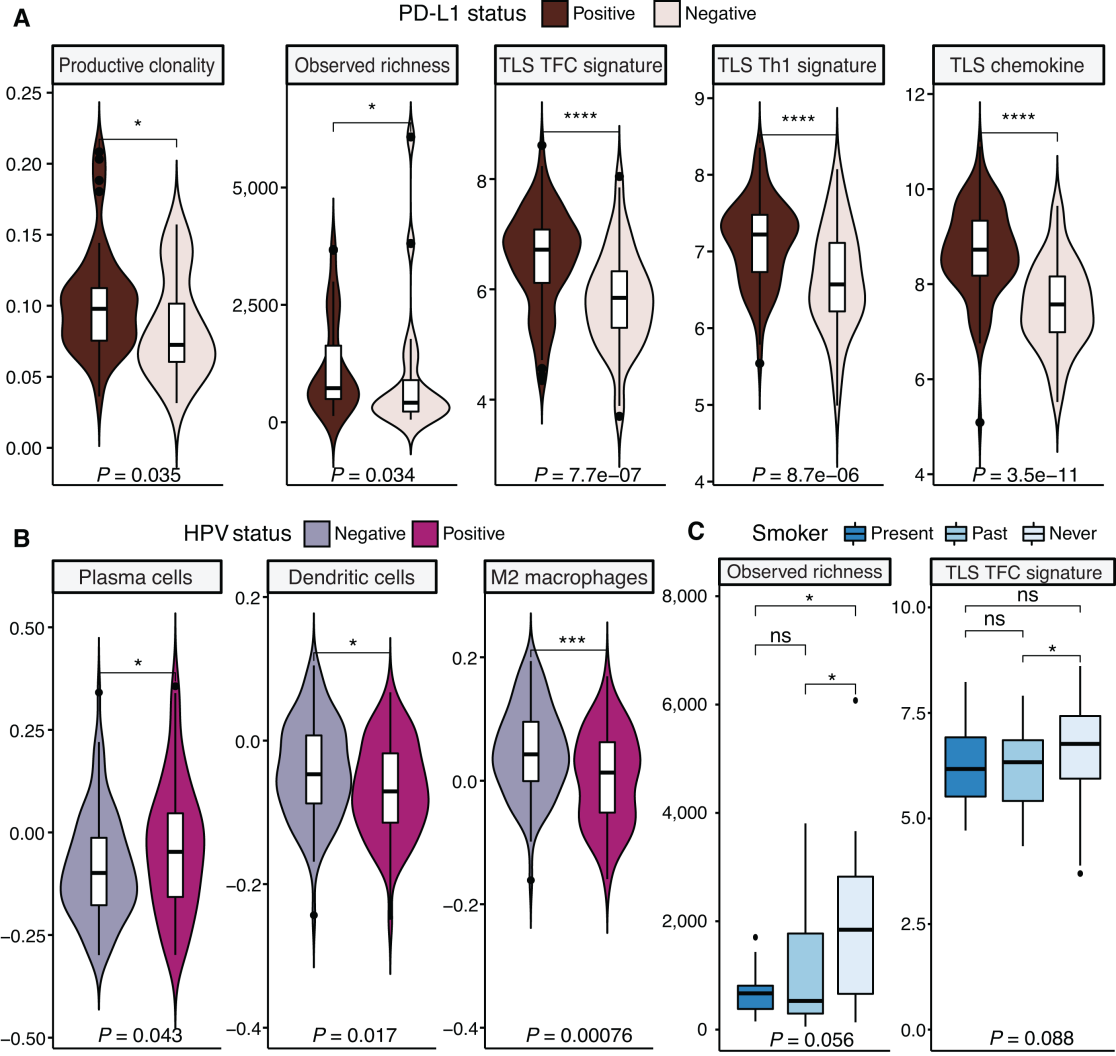
Links between risk factors of HNSCC, exhaustion, and TCR repertoire. **A,** PD-L1–positive patients display higher productive clonality, observed richness and TLS signature scores. **B,** HPV-positive tumors have higher PC and DC content as well as lower M2 macrophage infiltration. **C,** Never smokers display higher observed richness of the TCR repertoires and stronger TLS TFC signals. ****, *P* < 0.0001; ***, *P* < 0.001; **, *P* < 0.01; *, *P* < 0.05; ns, nonsignificant (*P* > 0.05).

As already demonstrated in the previous sections, the patients’ demographic characteristics, behavioral and clinical histories may impact their cancer trajectory in relation to immune responses. We explored further how these parameters link to TME composition, exhaustion, and TLS formation, as determinants of immune “hot” phenotypes. As expected, PD-L1–positive patients demonstrated higher TCR productive clonality and observed richness, as well as markedly higher TLS signature scores (Fig. 5A).

HPV status is a major determinant of outcome in HNSCC, with HPV-positive samples showing overall better survival (52, 53). Besides being enriched in the PC high subgroup, HPV-positive tumors were also found to present a higher plasma and DC content (in line with the former observation) and, interestingly, a decreased abundance of M2 macrophages (Fig. 5B), suggesting a specific immune priming of tumors in the context of HPV infection as demonstrated in previous studies (54, 55).

The smoking history of the patients was significantly associated with features of the TCR repertoire and TLS TFC signaling. As such, never smokers (19% of the cohort) had a greater observed TCR richness and TLS TFC score (Fig. 5C), which may be related to the significantly improved overall survival over present smokers (Supplementary Table S3) and highlights the potential impact of smoking on the ability to sustain a diverse immune response. No significant TCR repertoire differences were observed on the basis of TLS Th1 and chemokine scores.

## Discussion

**FIGURE 6:**
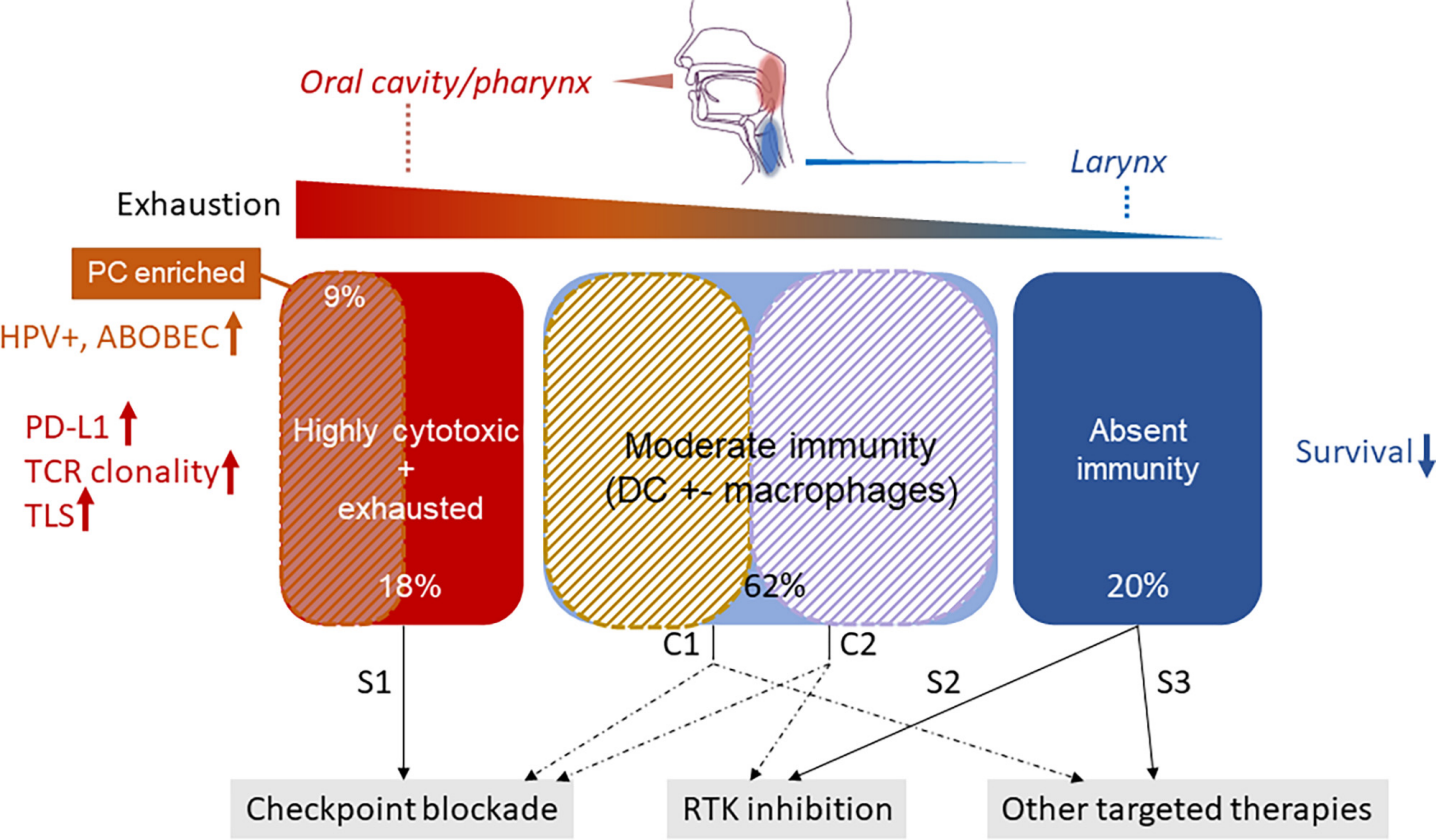
The immune ecosystem of HNSCC and suggested therapeutic strategies. Two extremes of tumor immunity are observed: highly cytotoxic and exhausted and immune-excluded, with specific genomic changes and immune cell enrichment. 9% of the cytotoxic tumors are enriched in PCs, with a dominance of HPV positivity and increased APOBEC activity. Tumor location impacts the developing immunity, with oral cavity and pharynx hosting an increased cytotoxic immune make-up. Exhausted groups may be amenable to checkpoint blockade, while immune-excluded ones may be subject to growth signaling suppression via tyrosine kinase inhibition. The moderate immunity group would likely benefit from combination therapies targeting both tumor intrinsic pathways (tyrosine kinase signaling) and immune reactivation via checkpoint blockade. RTK = receptor tyrosine kinase; TLS = tertiary lymphoid structures; TCR = T-cell repertoire; PC = plasma cell; DC = dendritic cell. S1–3: single-agent therapies; C1–2: combination therapies.

The emerging immune landscape of HNSCC primary disease before treatment is split on a spectrum between “hot” and “cold” immunity that is strongly shaped by tumor etiology and suggests different therapeutic strategies (Fig. 6). On the one side, we see three subgroups enriched in cytotoxic, plasma or DCs, with higher TCR diversity and exhaustion (total of ∼43% of patients), which could possibly benefit from immunotherapy. 9% of these patients have increased PC content and APOBEC activity, which has been shown to generate heteroclitic neoepitopes that activate T-cell responses (56). Interestingly, the majority of these patients are HPV positive, and this group has been linked with higher immunity in previous TCGA studies too (57). This poses an intriguing hypothesis that the viral load may be triggering the activity of the antiviral cytidine deaminase APOBEC, which in turn leads to accumulation of antigenic mutations. Furthermore, these associations confirm findings on enriched germinal center B-cell signatures and TLSs in HPV-positive head and neck tumors from Ruffin and colleagues (58).

The rest of the patients show mixed immune infiltrates with high macrophage load and in a considerable fraction of the cohort (∼20%) even complete absence of immune reactivity. Importantly, this latter group lacks biomarkers generally associated with clinical benefit on immune checkpoint blockade and shows the worst overall survival outcome. It will be important to further characterize these patients for the identification of potential targeted therapies or other combined approaches. In fact, we observe that the signaling of oncogenic kinases along the Ras/ERK and PI3K/AKT pathways is not independent of tumor immunity in HNSCC, suggesting that targeting some of these components may be a viable option to suppress tumor growth or to reactivate immune responses. Emerging evidence is in fact suggesting that signaling through these pathways may be a more common and direct mechanism of immune modulation than previously anticipated in a variety of cancers (59). Dual targeting of PI3K and immune checkpoints has indeed been suggested to be potentially effective in several studies, including in HNSCC (60–62). This could be made possible by recent successes of PIK3CA inhibitors in a metastatic setting (63, 64), with several components of this pathway deemed as promising molecular targets in head and neck cancer (65, 66). Preclinical studies will be required to test these hypotheses and identify the exact patient segment that might benefit from such therapeutic strategies. A limitation of this current study is the lack of subsequent treatment information for these patients

Genomically, HNSCC tumors are dominated by mutations resulting through altered patterns of APOBEC activity, but the landscape of mutational drivers is overall highly heterogeneous, similarly to that reported by previous TCGA studies, with TP53 and CDKN2A as major drivers (4). While a targeted approach could be effective in the subset of patients with mutations activating kinase signaling, as previously proposed by studies of TCGA cohort (4), this seems to still be a minority and it is likely that a complementary strategy aiming to reactivate immune responses could be effective in a larger fraction of patients. Given the increase in APOBEC activity in PC rich tumors, these cases may also respond to combination therapies involving checkpoint blockade and APOBEC inhibitors (67, 68). These hypotheses need further investigation in suitable *in vivo* models.

A limitation of the study is the ethnic composition of the cohort, which is predominantly Caucasian and African American patients with limited representation of Asian and Hispanic patients, and is therefore challenging to report important differences in molecular features of HNSCC across ethnicities. However, we find that the HNSCC site of occurrence and the patients’ histories of smoking behavior may impact T-cell responses by modulating TCR profiles. These are easily measurable criteria that could be used to inform patient selection in view of immunotherapy in addition to the classical markers currently used in the clinic. Studies in larger, better powered, and more ethnically diverse cohorts will be needed to validate these potential prognostic biomarkers in HNSCC. Additional profiling and integration of structural variants or epigenetic changes will also be essential to further understand the evolution of this disease, and could better delineate the drivers of the lesser characterized group of HPV-negative cancers.

## Supplementary Material

Supplementary Tables 1-3 Supplementary Table 1 shows Clinical characteristics of the cohort (n=162). Supplementary Table 2 shows TLS signatures employed in the analysis. Supplementary Table 3 shows Multivariate Cox Proportional Hazards analysis for the HNSCC cohort.

Supplementary Figures 1-23 Supplementary Figure 1 shows differences in immune cell infiltration/activity between the five immunity groups. Supplementary Figure 2 shows correlation in inferred cell abundance between distinct immune cell subpopulations in the TME of HNSCC tumours. Supplementary Figure 3 shows validation of immune phenotypes in n=520 HNSCC TCGA samples. Supplementary Figure 4 shows PD-L1 expression in TCGA immunity groups. Supplementary Figure 5 shows tumour microenvironment landscapes by HPV status. Supplementary Figure 6 shows differences in immune cell infiltration by HNSCC tumour sites of origin. Supplementary Figure 7 shows overall survival differences by immunity subgroup in the discovery cohort. Supplementary Figure 8 shows overall survival differences by immunity subgroup in the TCGA cohort (n=518). Supplementary Figure 9 shows differences in total mutational burden (log 10 scale) between the five immunity subgroups. Supplementary Figure 10 shows the tumour mutational burden is inversely correlated with the observed richness of the TCR repertoire. Supplementary Figure 11 shows exhaustion and TCR repertoire variation in relation to subclonality. Supplementary Figure 12 shows the top prevalent signatures in the cohort, as inferred by deconstructSigs. Supplementary Figure 13s shows somatic mutations across the Ras/MAPK and PI3K/AKT kinase signalling pathway components. Supplementary Figure 14 shows correlation between the expression of genes in the Ras/MAPK and PI3K/AKT pathway and TCR productive clonality. Supplementary Figure 15 shows correlation between the expression of genes in the Ras/MAPK and PI3K/AKT pathway and the observed richness of the TCR repertoire. Supplementary Figure 16 shows expression of 29 (out of 52) receptor tyrosine kinases and downstream genes in the MAPK/ERK and PI3K/AKT pathways was measurable using the Nanostring gene expression panel. Supplementary Figure 17 shows validation of EGFR expression trends by immunity group in TCGA. Supplementary Figure 18 shows modelling the two immunity classes (high/low) based on signalling of all measurable genes across tyrosine kinase pathways. Supplementary Figure 19 shows expression of markers associated with high cytotoxicity across the immunity groups. Supplementary Figure 20 shows expression of TLS-specific signature genes and chemokine signalling by HPV status. Supplementary Figure 21 shows IHC protein staining for CD8+/CD3+ T cells and Treg markers (FOXP3, GITR) by HPV status. Supplementary Figure 22 shows validation of TLS signature trends in TCGA. Supplementary Figure 23 shows the TLS signature score is weakly correlated with the productive clonality of the TCR repertoire.
