# Supplementary material for: Immune Cell Abundance and T-cell Receptor Landscapes Suggest New Patient Stratification Strategies in Head and Neck Squamous Cell Carcinoma
Source: Cancer Res Commun. 2023 Oct 20;3(10):2133–45. doi: 10.1158/2767-9764.CRC-23-0155 (PMC10588680; doi:10.1158/2767-9764.CRC-23-0155)

**Supplementary Table 1. Clinical characteristics of the cohort (n=162)**

**
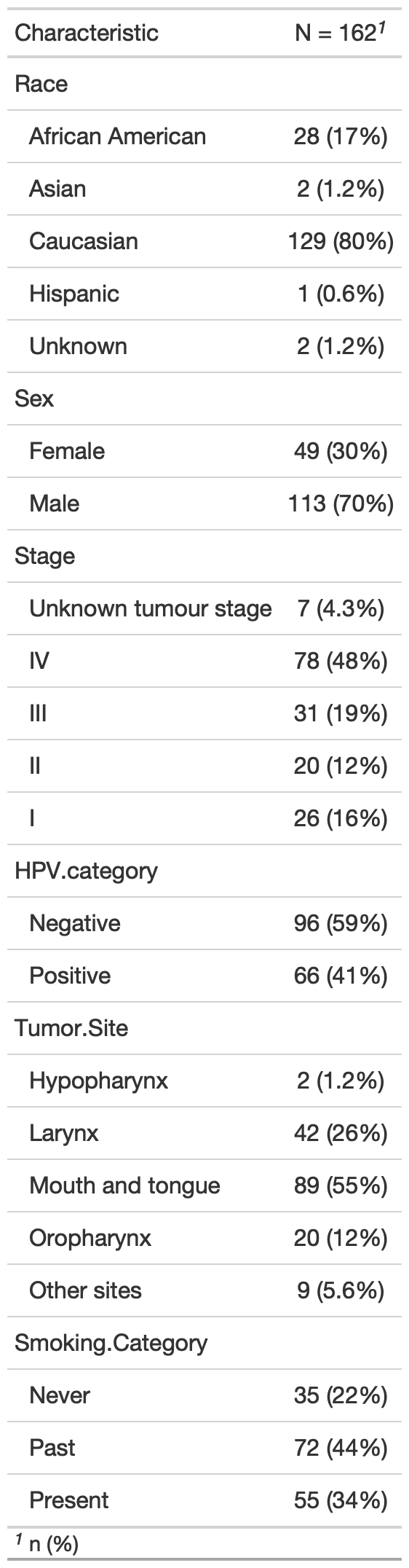
**

**Supplementary Table 2.** TLS signatures employed in the analysis.

| TLS TFH signature | TLS Th1 signature | TLS chemokine signature |
| --- | --- | --- |
| CXCL13 | CD4 | CXCL13 |
| CD200 | CCR5 | CXCL11 |
| FBLN7 | CXCR3 | CXCL10 |
| ICOS | CSF2 | CXCL9 |
| SGPP2 | IGSF6 | CCL21 |
| SH2D1A | IL2RA | CCL19 |
| TIGIT | CD38 | CCL18 |
| PDCD1 | CD40 | CCL8 |
|  | CD5 | CCL5 |
|  | MS4A1 | CCL4 |
|  | SDC1 | CCL3 |
|  | GFI1 | CCL2 |
|  | IL1R1 |  |
|  | IL1R2 |  |
|  | IL10 |  |
|  | CCL20 |  |
|  | IRF4 |  |
|  | TRAF6 |  |
|  | STAT5A |  |

**Supplementary Table 3. Multivariate Cox Proportional Hazards analysis for the HNSCC cohort.** The overall survival is modelled based on clinical and TME subgroup data. Global p-value = 0.003, AIC = 702.77, concordance index = 0.71.


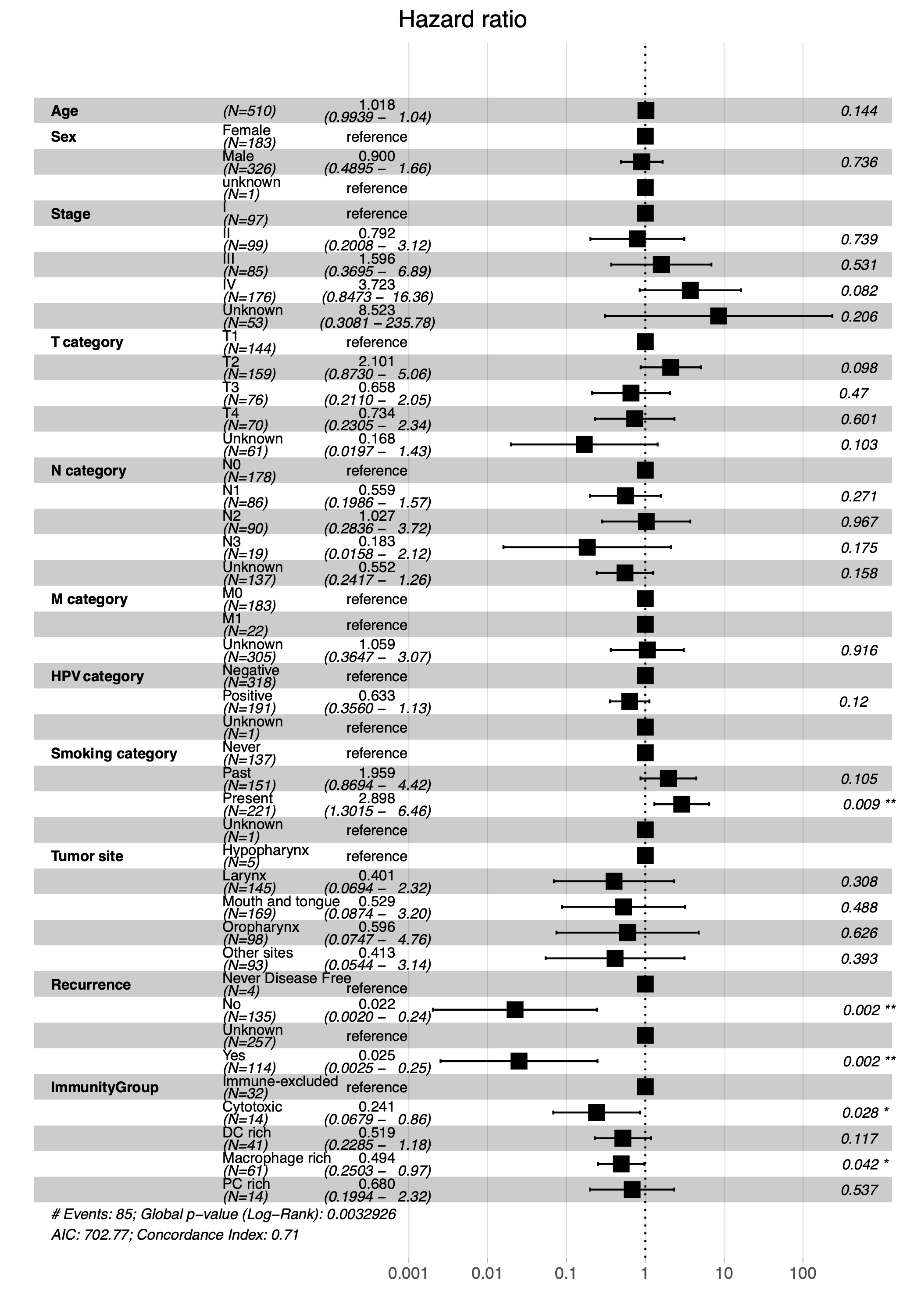

Supplement: Supplementary Tables 1-3 — Supplementary Table 1 shows Clinical characteristics of the cohort (n=162). Supplementary Table 2 shows TLS signatures employed in the analysis. Supplementary Table 3 shows Multivariate Cox Proportional Hazards analysis for the HNSCC cohort. [file crc-23-0155-s01.docx]
