# Supplementary material for: Immune Cell Abundance and T-cell Receptor Landscapes Suggest New Patient Stratification Strategies in Head and Neck Squamous Cell Carcinoma
Source: Cancer Res Commun. 2023 Oct 20;3(10):2133–45. doi: 10.1158/2767-9764.CRC-23-0155 (PMC10588680; doi:10.1158/2767-9764.CRC-23-0155)
Supplement: Supplementary Figures 1-23 — Supplementary Figure 1 shows differences in immune cell infiltration/activity between the five immunity groups. Supplementary Figure 2 shows correlation in inferred cell abundance between distinct immune cell subpopulations in the TME of HNSCC tumours. Supplementary Figure 3 shows validation of immune phenotypes in n=520 HNSCC TCGA samples. Supplementary Figure 4 shows PD-L1 expression in TCGA immunity groups. Supplementary Figure 5 shows tumour microenvironment landscapes by HPV status. Supplementary Figure 6 shows differences in immune cell infiltration by HNSCC tumour sites of origin. Supplementary Figure 7 shows overall survival differences by immunity subgroup in the discovery cohort. Supplementary Figure 8 shows overall survival differences by immunity subgroup in the TCGA cohort (n=518). Supplementary Figure 9 shows differences in total mutational burden (log 10 scale) between the five immunity subgroups. Supplementary Figure 10 shows the tumour mutational burden is inversely correlated with the observed richness of the TCR repertoire. Supplementary Figure 11 shows exhaustion and TCR repertoire variation in relation to subclonality. Supplementary Figure 12 shows the top prevalent signatures in the cohort, as inferred by deconstructSigs. Supplementary Figure 13s shows somatic mutations across the Ras/MAPK and PI3K/AKT kinase signalling pathway components. Supplementary Figure 14 shows correlation between the expression of genes in the Ras/MAPK and PI3K/AKT pathway and TCR productive clonality. Supplementary Figure 15 shows correlation between the expression of genes in the Ras/MAPK and PI3K/AKT pathway and the observed richness of the TCR repertoire. Supplementary Figure 16 shows expression of 29 (out of 52) receptor tyrosine kinases and downstream genes in the MAPK/ERK and PI3K/AKT pathways was measurable using the Nanostring gene expression panel. Supplementary Figure 17 shows validation of EGFR expression trends by immunity group in TCGA. Supplementary Figu [file crc-23-0155-s02.docx]

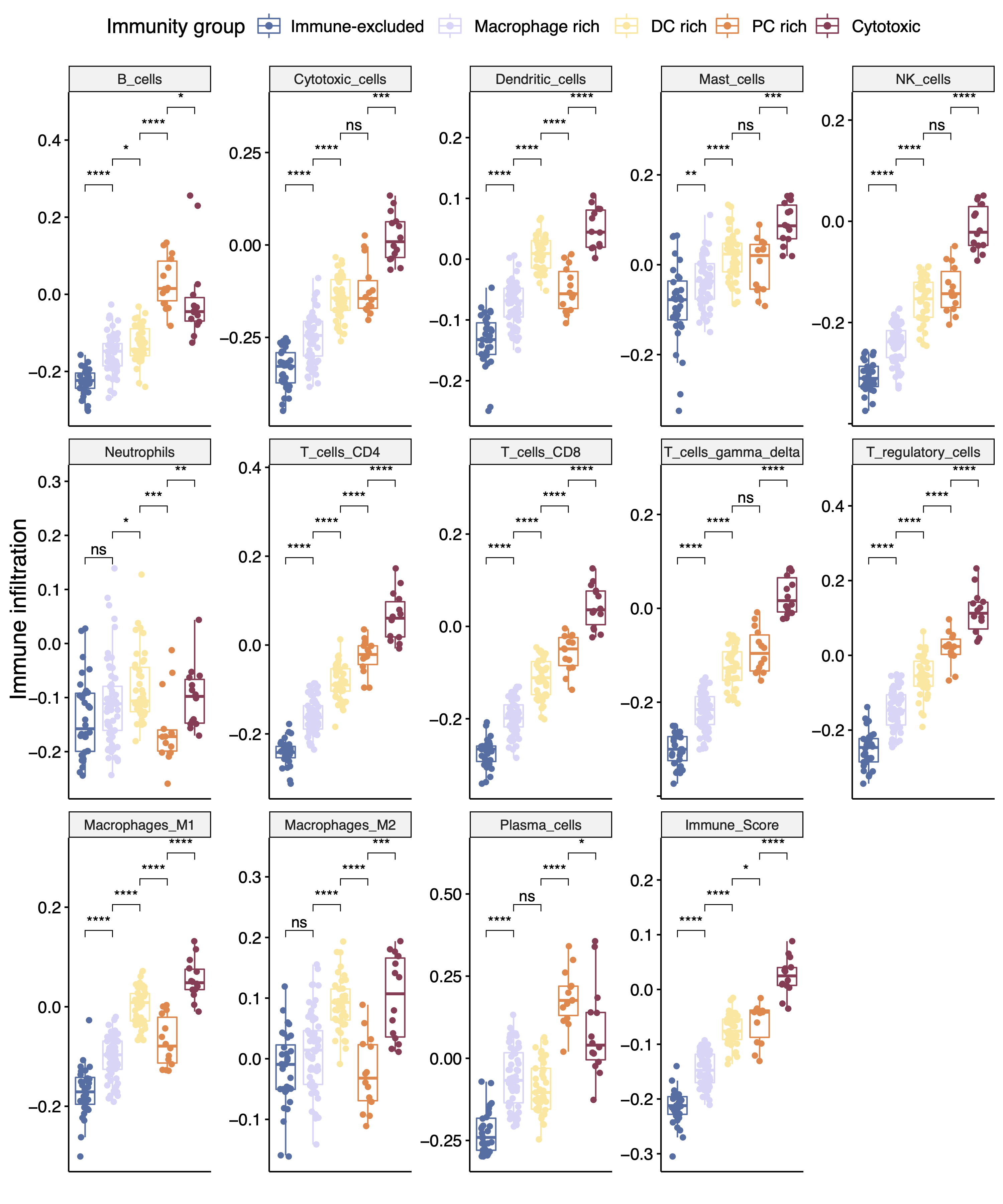


**Supplementary Figure 1. Differences in immune cell infiltration/activity between the five immunity groups.** **** p<0.0001; *** p<0.001; ** p<0.01; * p<0.05; ns – non-significant (p>0.05).


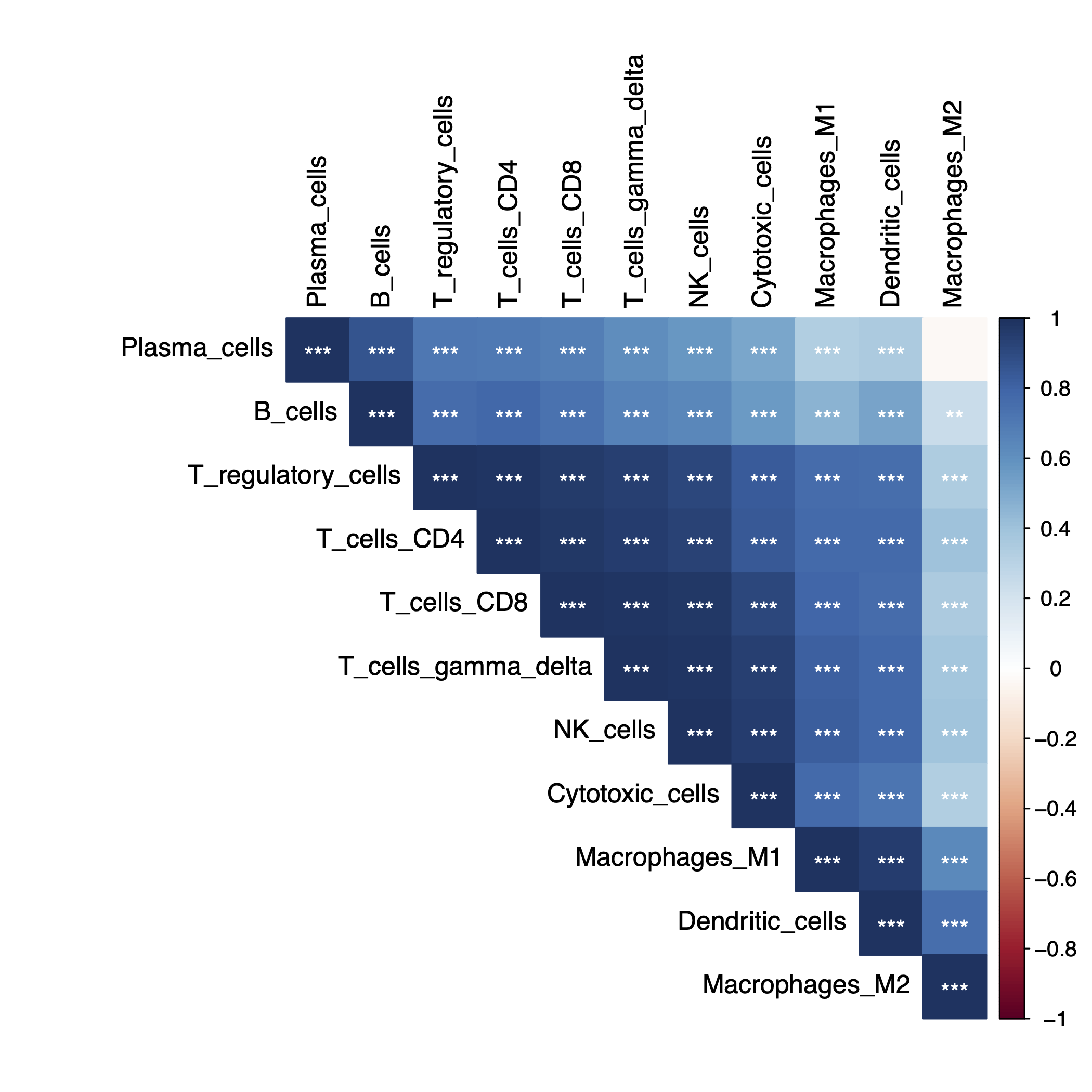


**Supplementary Figure 2. Correlation in inferred cell abundance between distinct immune cell subpopulations in the TME of HNSCC tumours.** The colour gradient reflects the Pearson correlation coefficient. *** p<0.001.


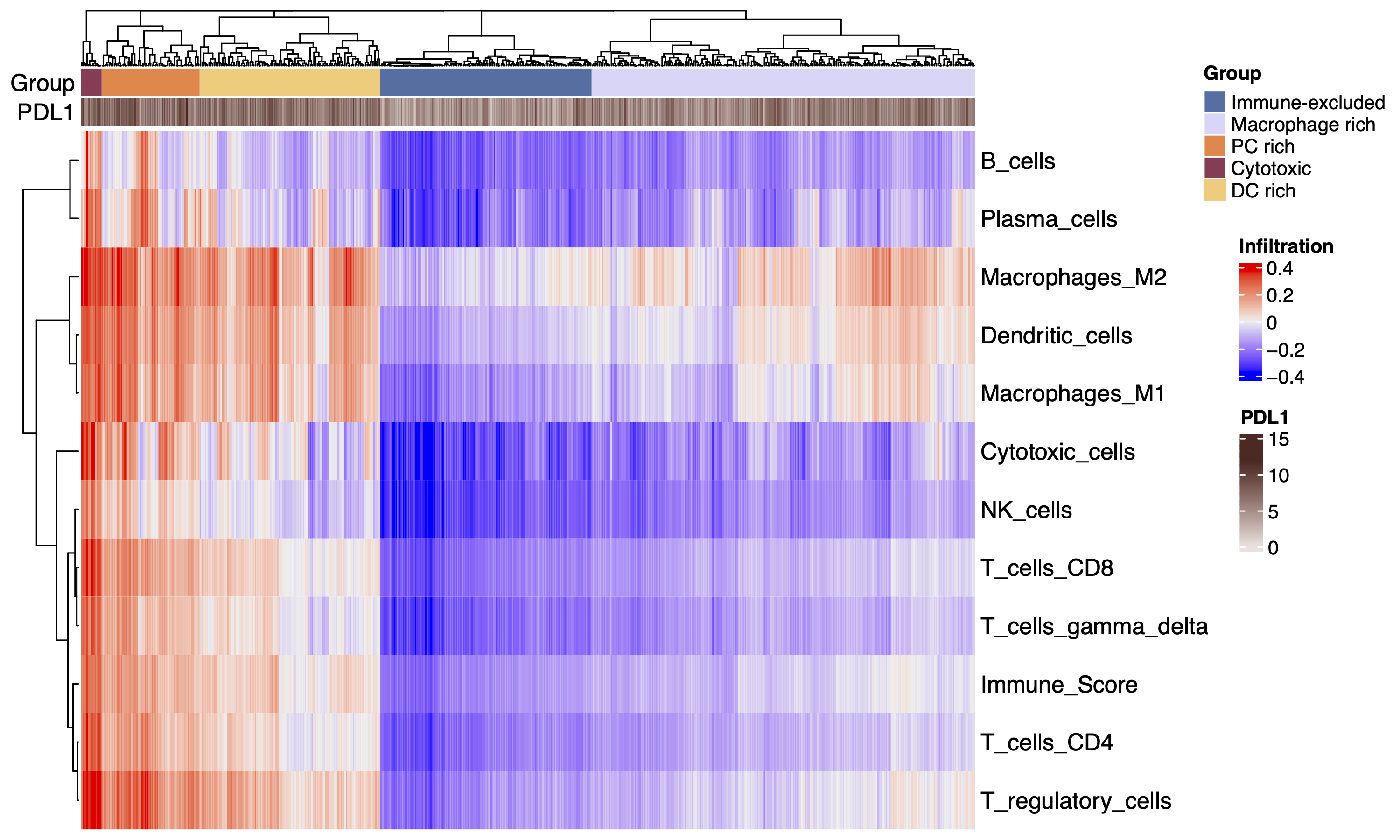


**Supplementary Figure 3. Validation of immune phenotypes in n=520 HNSCC TCGA samples.** Samples (columns) are clustered according to their estimated immune composition (rows) from bulk RNA-seq data by ConsensusTME. Hierarchical clustering yields five subgroups which have similar features to the ones in the discovery cohort and have been annotated as such. The PD-L1 expression is also annotated and appears increased in the groups with higher cytotoxicity.


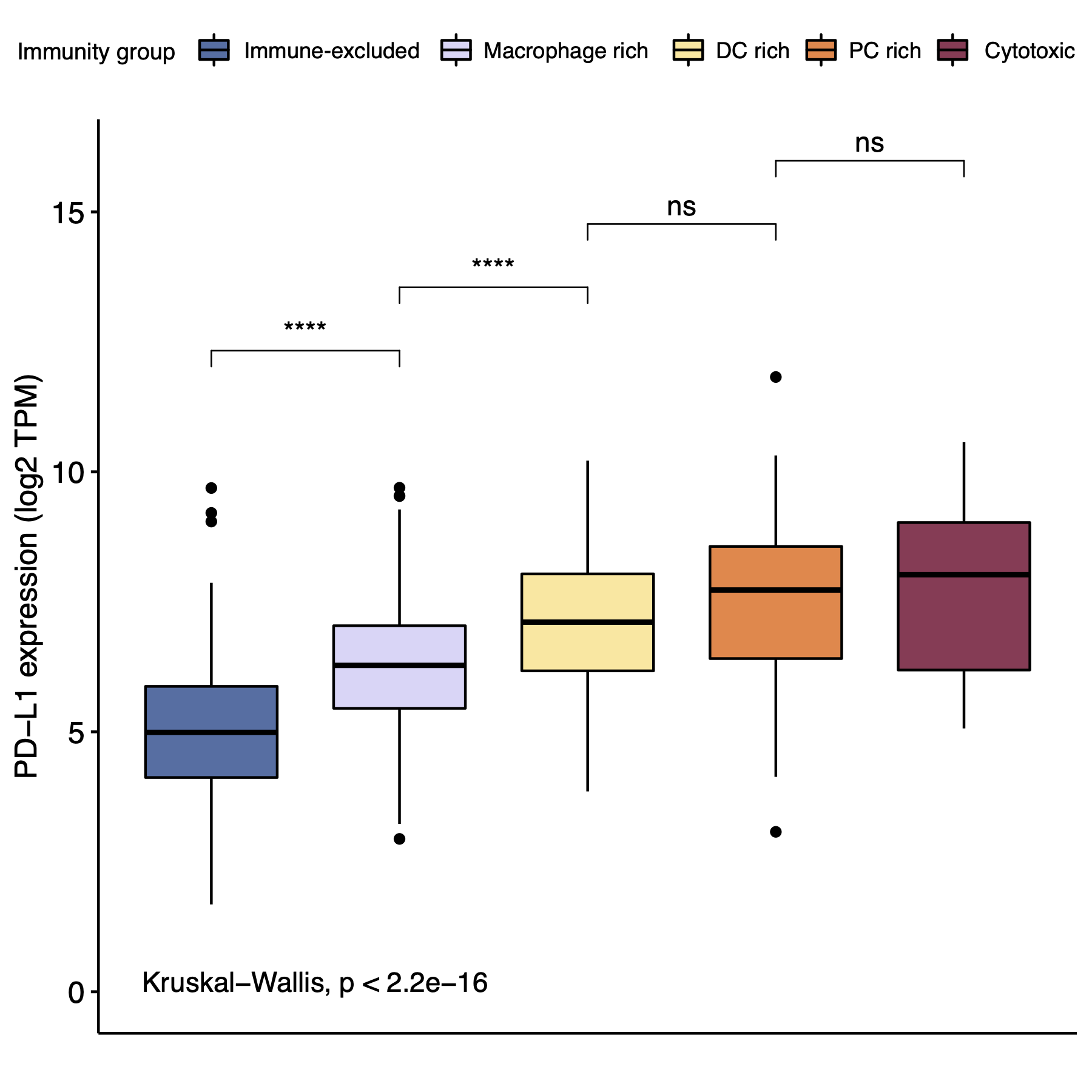


**Supplementary Figure 4. PD-L1 expression in TCGA immunity groups.** The expression of PD-L1 is significantly different between the different groups and increases from low to high immunity similarly to the discovery cohort. **** p<0.00001; *** p<0.0001; ns – non-significant (p>0.05).


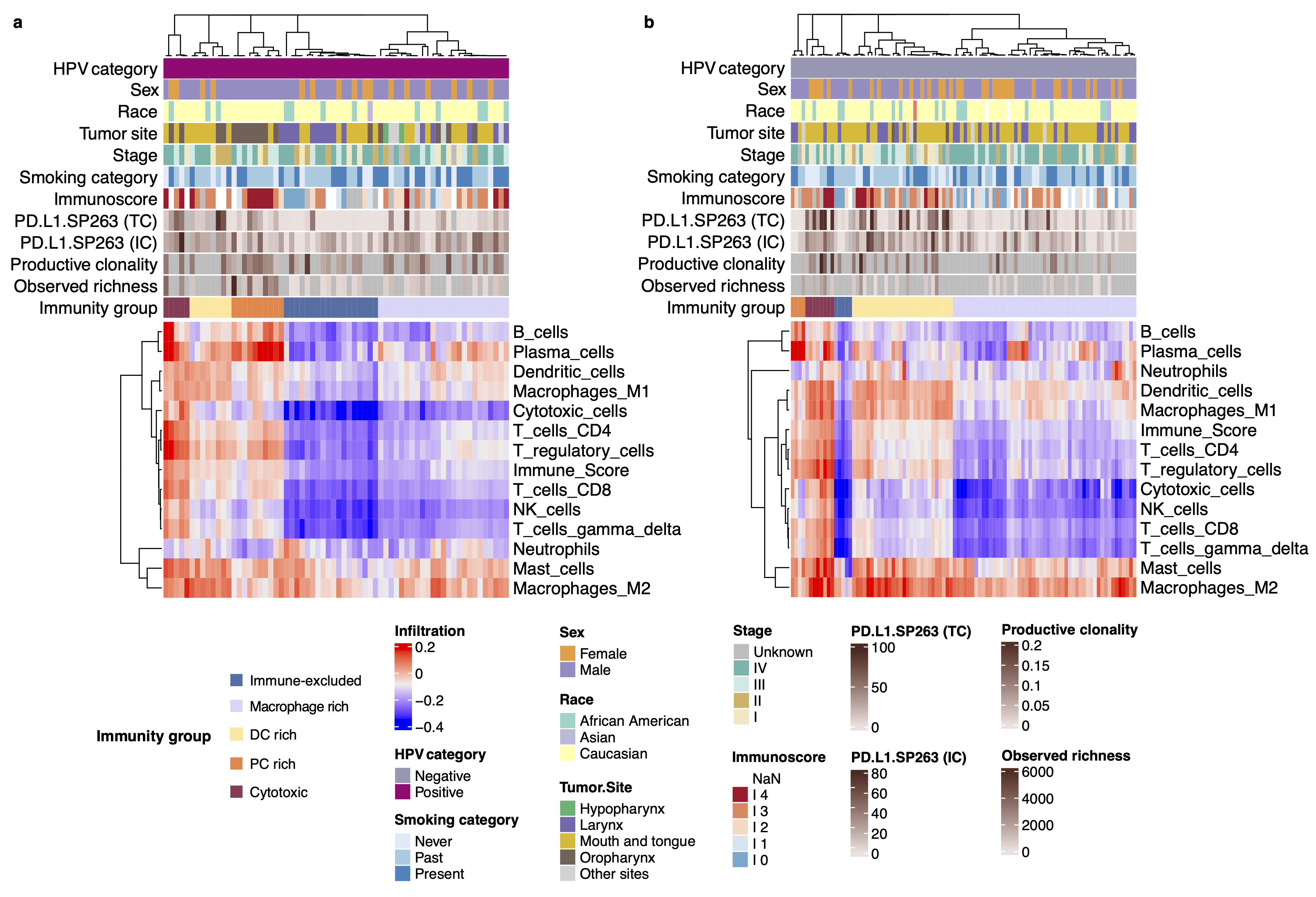


**Supplementary Figure 5. Tumour microenvironment landscapes** **by HPV status.** The heat maps display the immune cell enrichment/depletion in (a) HPV positive cancers and (b) HPV negative cancers, as inferred from the expression of cell-type specific markers using ConsensusTME and ssGSEA. Every column corresponds to a sample and every row depicts an immune subpopulation. Samples are clustered based on the predicted enrichment/depletion of immune cell populations. Relevant clinical features, Immunoscore (CD3 and CD8 quantification), PD-L1 staining by immunohistochemistry (TC - tumour cell and IC - immune cell positivity) and T cell receptor (TCR) repertoire characteristics (productive clonality, observed richness) are annotated. The same five subgroups as in the aggregated analysis are identified: cytotoxic (n=5 in HPV positive, n=8 in HPV negative), PC rich (n=10 in HPV positive, n=4 in HPV negative), DC rich (n=8 in HPV positive, n=28 in HPV negative), macrophage rich (n=25 in HPV positive, n=51 in HPV negative) and immune-excluded (n=18 in HPV positive, n=5 in HPV negative). Key clinical characteristics are also annotated.


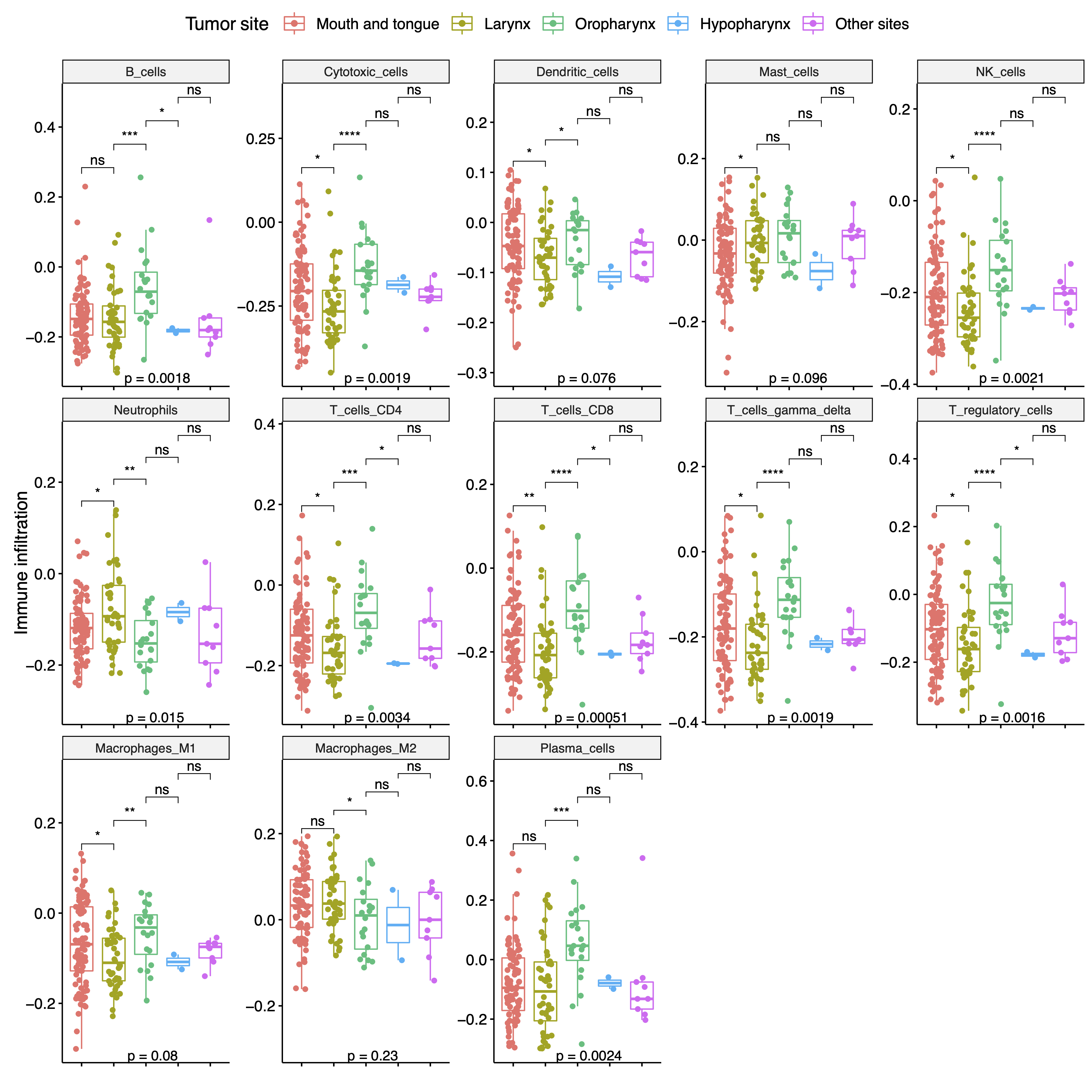


**Supplementary Figure 6.** Differences in immune cell infiltration by HNSCC tumour sites of origin. The immune score estimated by ConsensusTME is calculated as the ssGSEA score across the expression of all immune-related genes from every other category, excluding stromal cells. **** p<0.0001; *** p<0.001; ** p<0.01; * p<0.05; ns – non-significant (p>0.05).


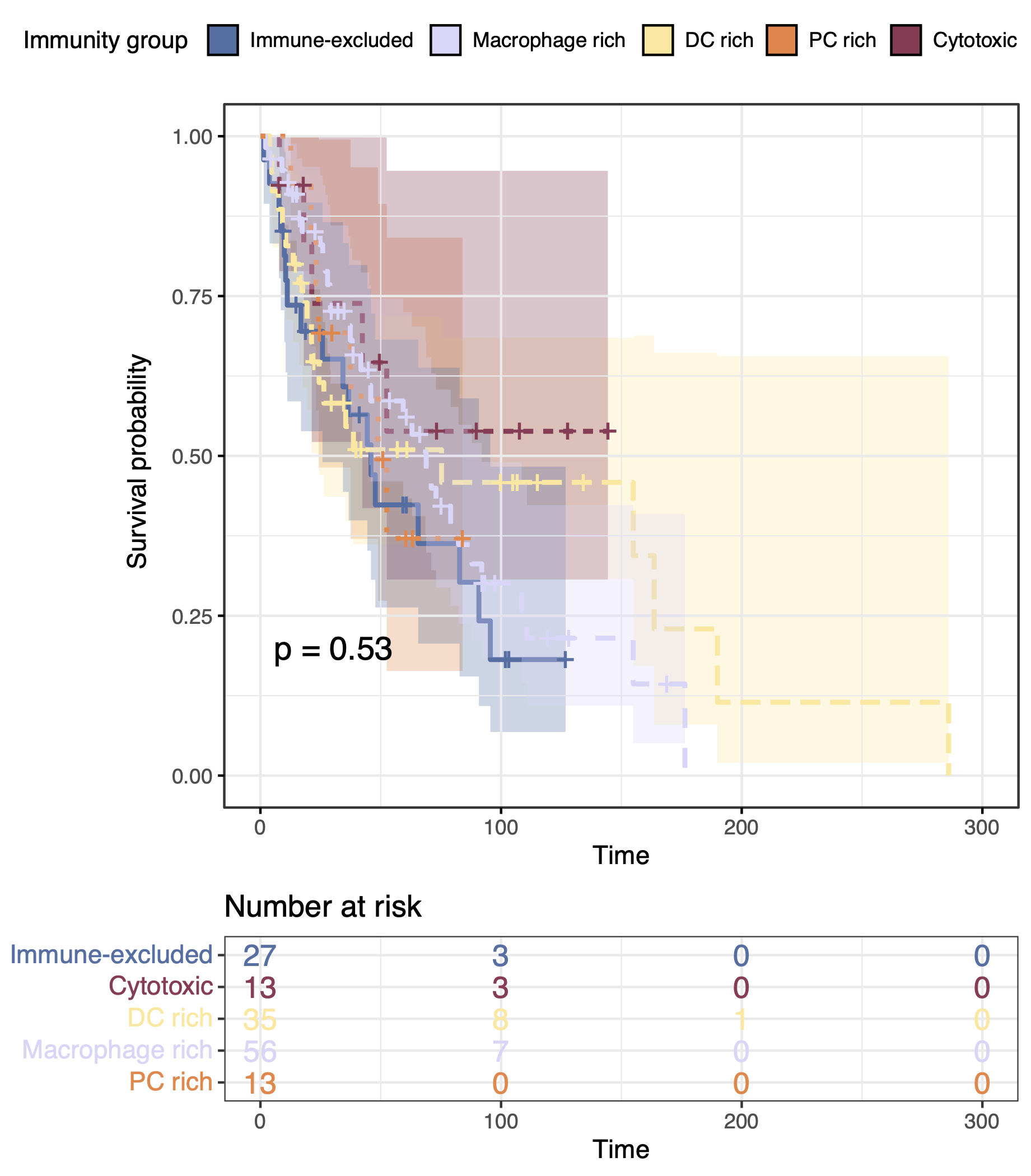


**Supplementary Figure 7. Overall survival differences by immunity subgroup in the discovery cohort.** The immune-excluded and macrophage rich groups have the worst survival, while the cytototoxic and DC rich groups have the most favourable outcomes (not accounting for other confounders).


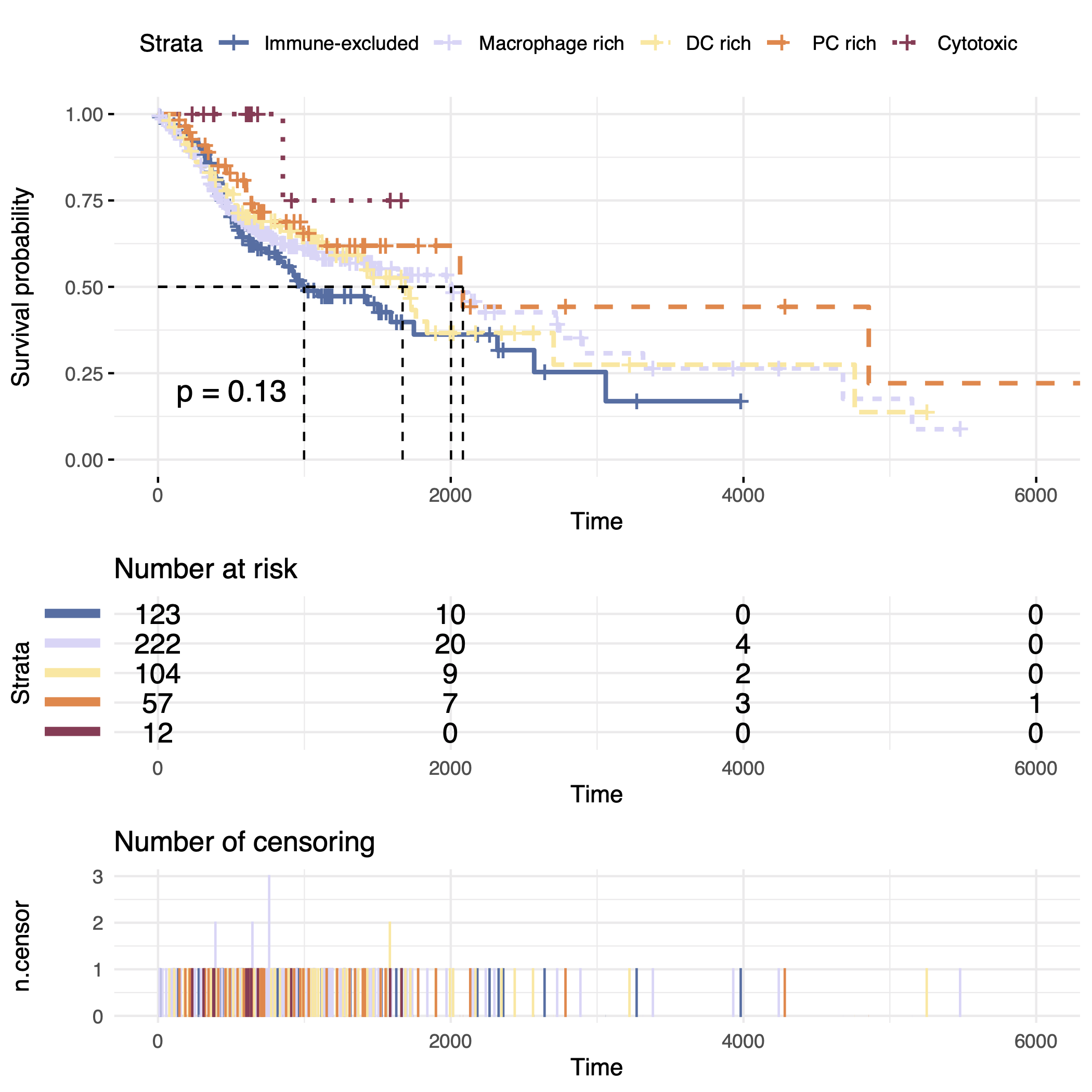


**Supplementary Figure 8. Overall survival differences by immunity subgroup in the TCGA cohort (n=518).** Differences in survival between the five defined immunity subgroups are shown. Similar trends as in the discovery cohort can be observed, but no significant differences.


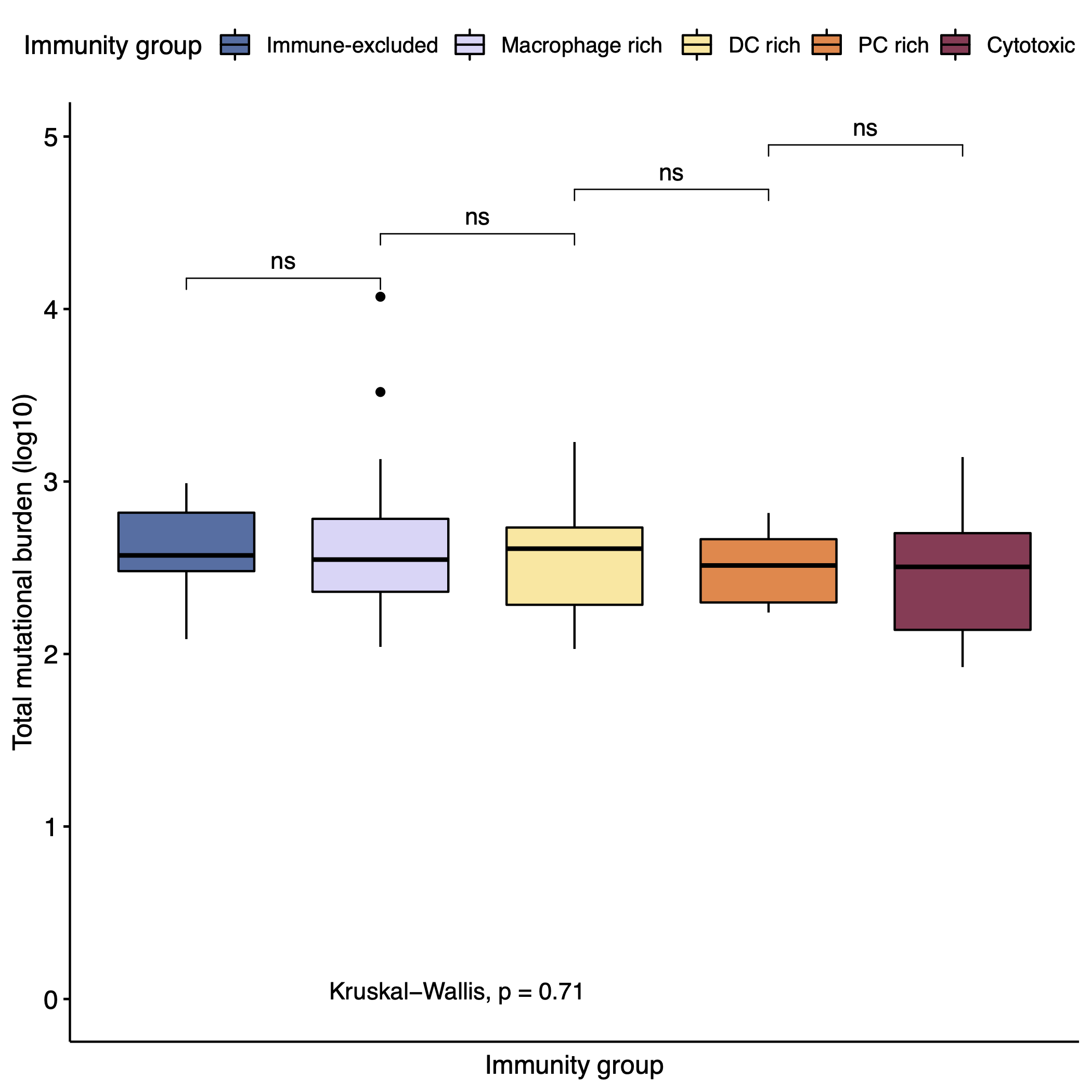


**Supplementary Figure 9.** Differences in total mutational burden (log 10 scale) between the five immunity subgroups. ns – non-significant (p>0.05).


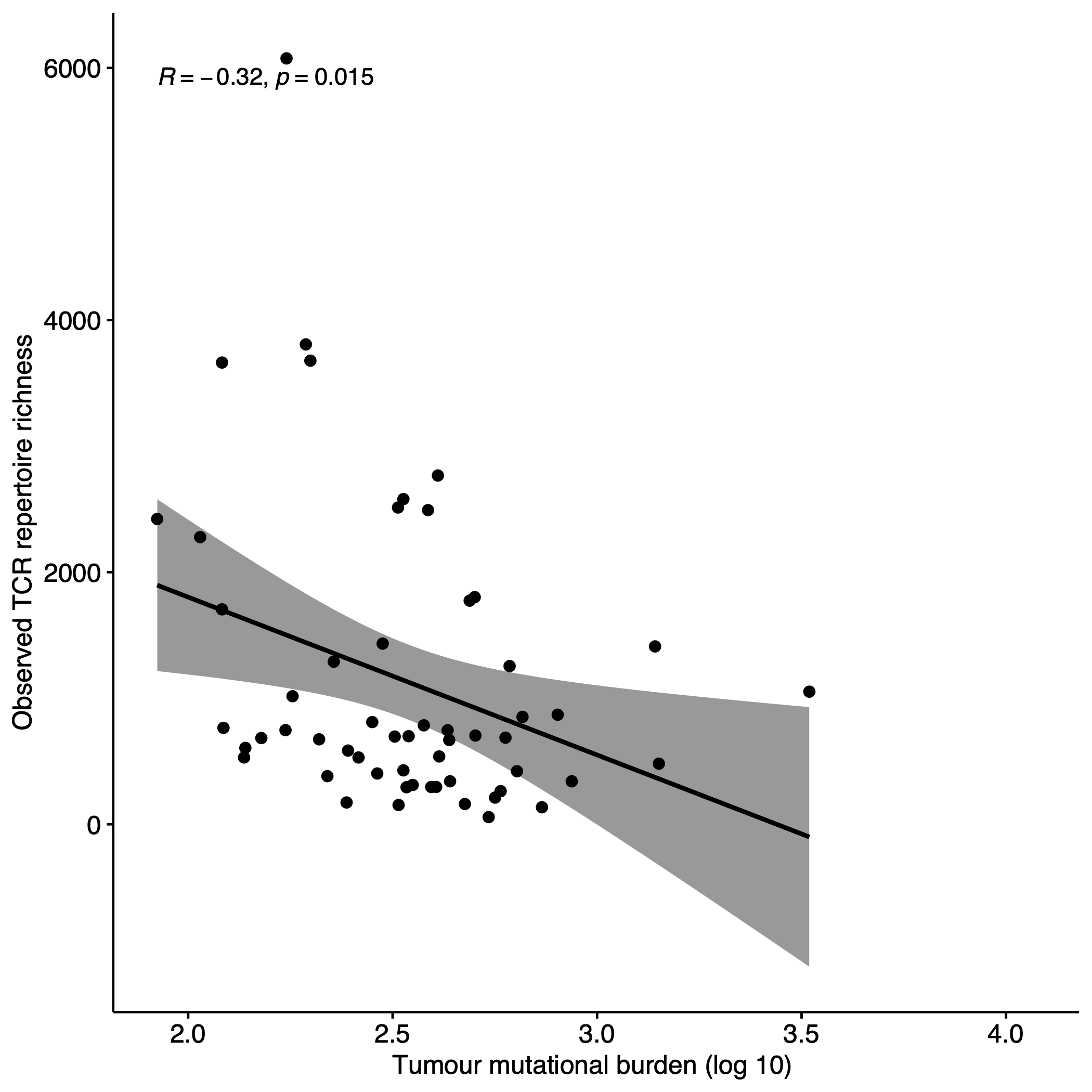


**Supplementary Figure 10.** The tumour mutational burden is inversely correlated with the observed richness of the TCR repertoire.


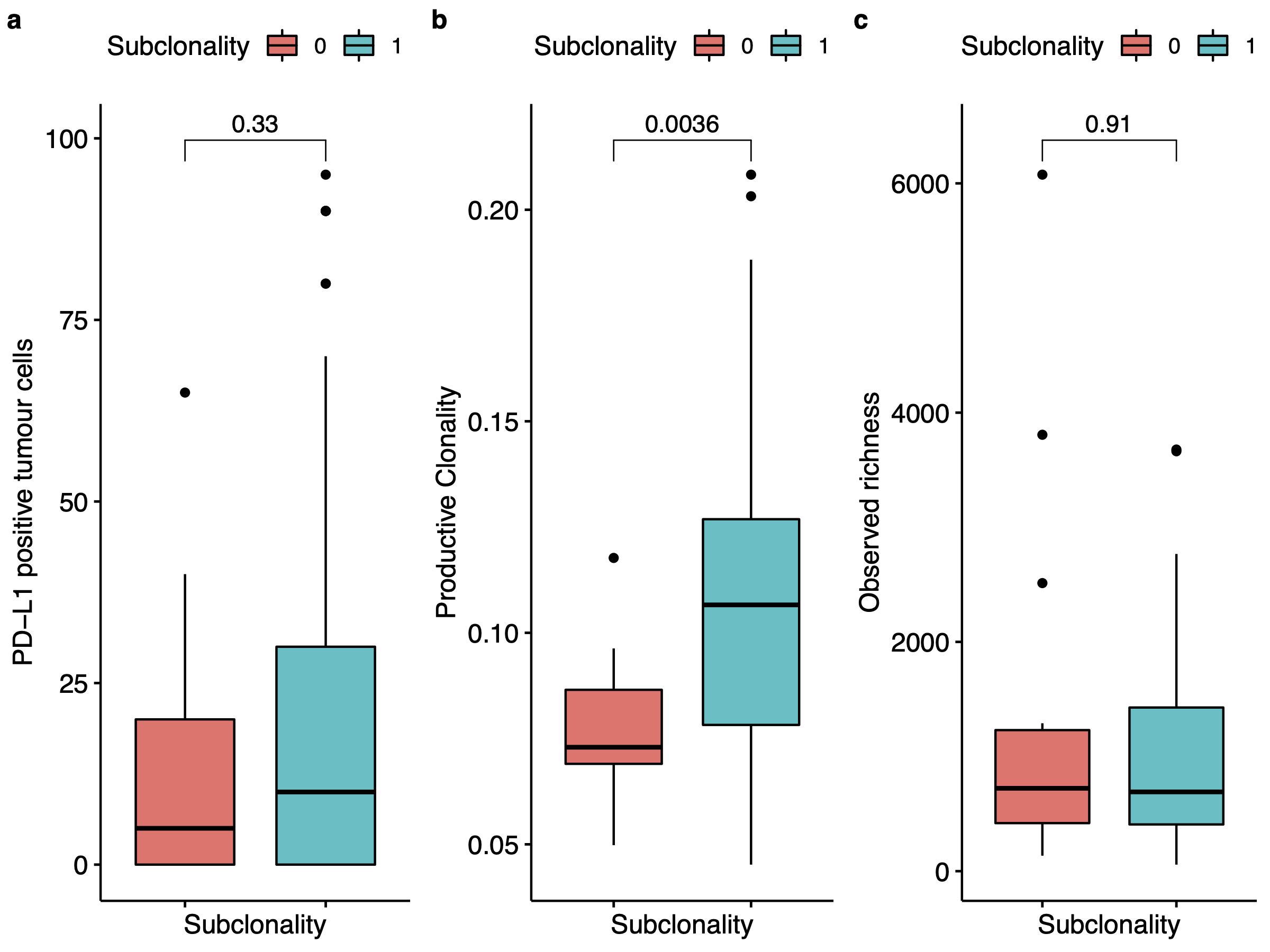


**Supplementary Figure 11.**  Exhaustion and TCR repertoire variation in relation to subclonality: (a) PD-L1 staining; (b) TCR productive clonality and (c) TCR observed richness are compared between samples with (1) and without (0) evidence of tumour subclonality.


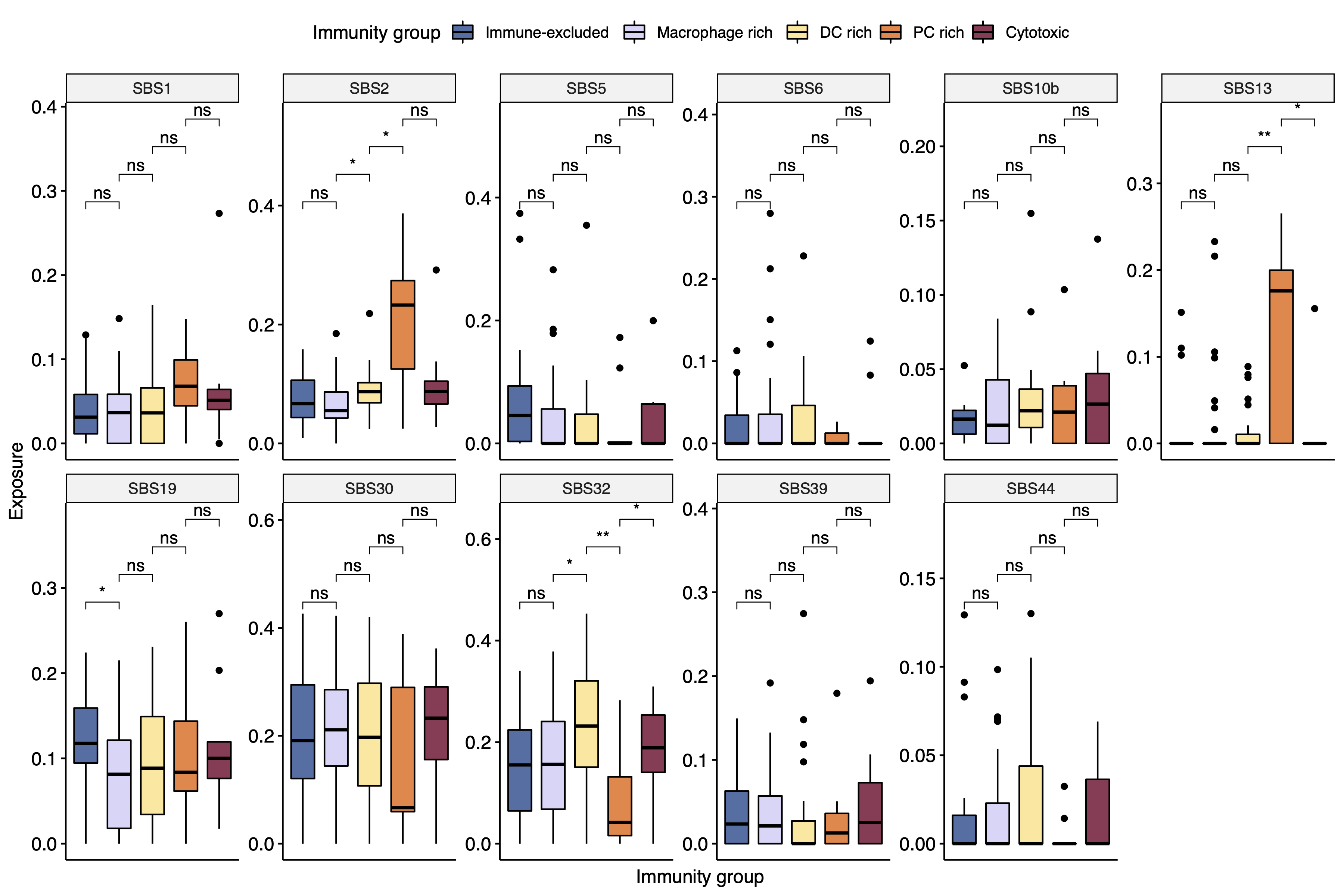


**Supplementary Figure 12.**  The top prevalent signatures in the cohort, as inferred by deconstructSigs. The relative contribution of each signature to the genomes of patients belonging to the five distinct immunity groups is shown. Kruskal-Wallis test p-values comparing the five groups are displayed underneath the plots. ** p<0.001; * p<0.05; ns – non-significant (p>0.05).


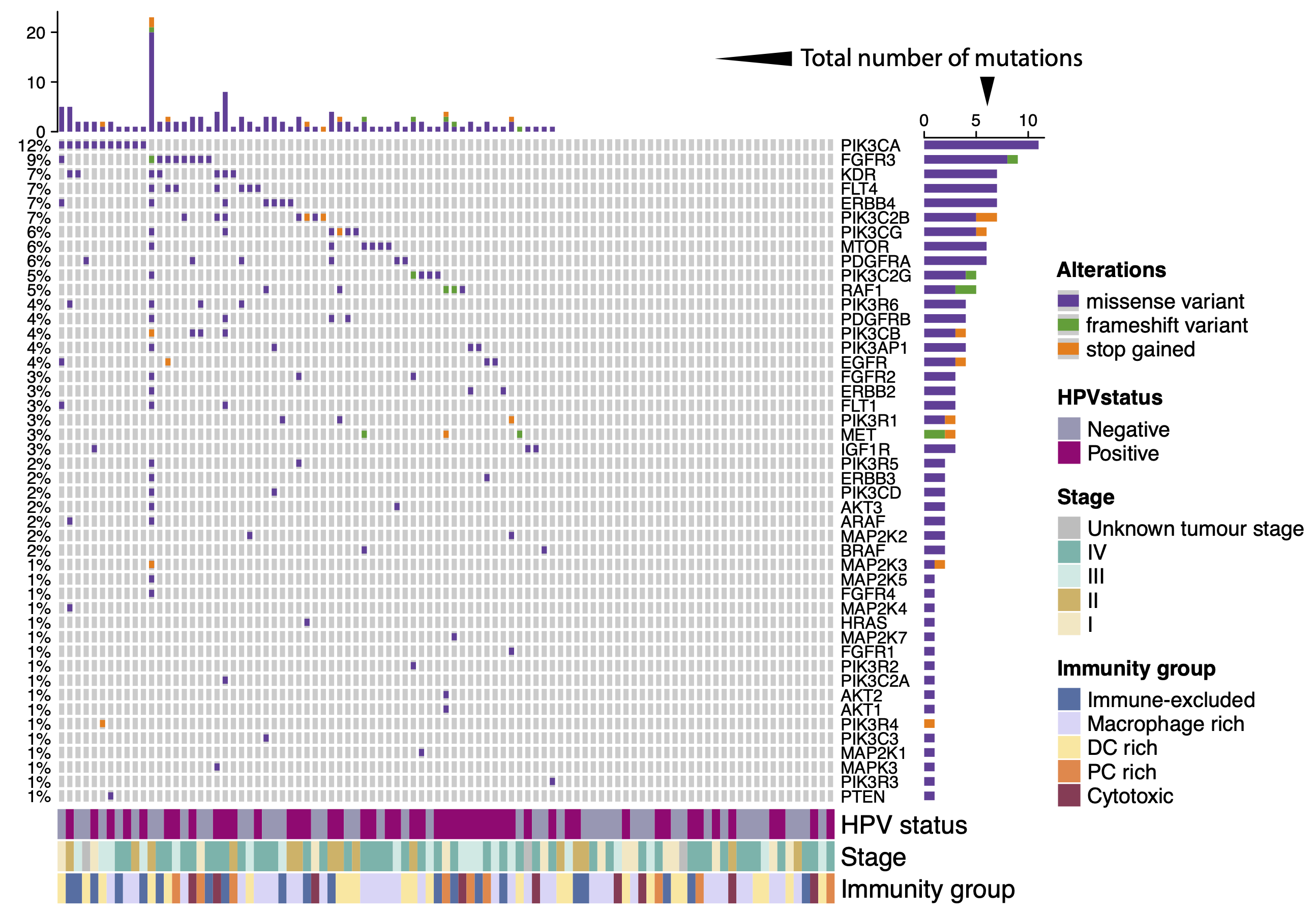


**Supplementary Figure 13.**  Somatic mutations across the Ras/MAPK and PI3K/AKT kinase signalling pathway components. The bar plots display the total number of mutations per gene (rows) and sample (columns), respectively, across the cohort. Genes with mutations in at least 2 patients are depicted.


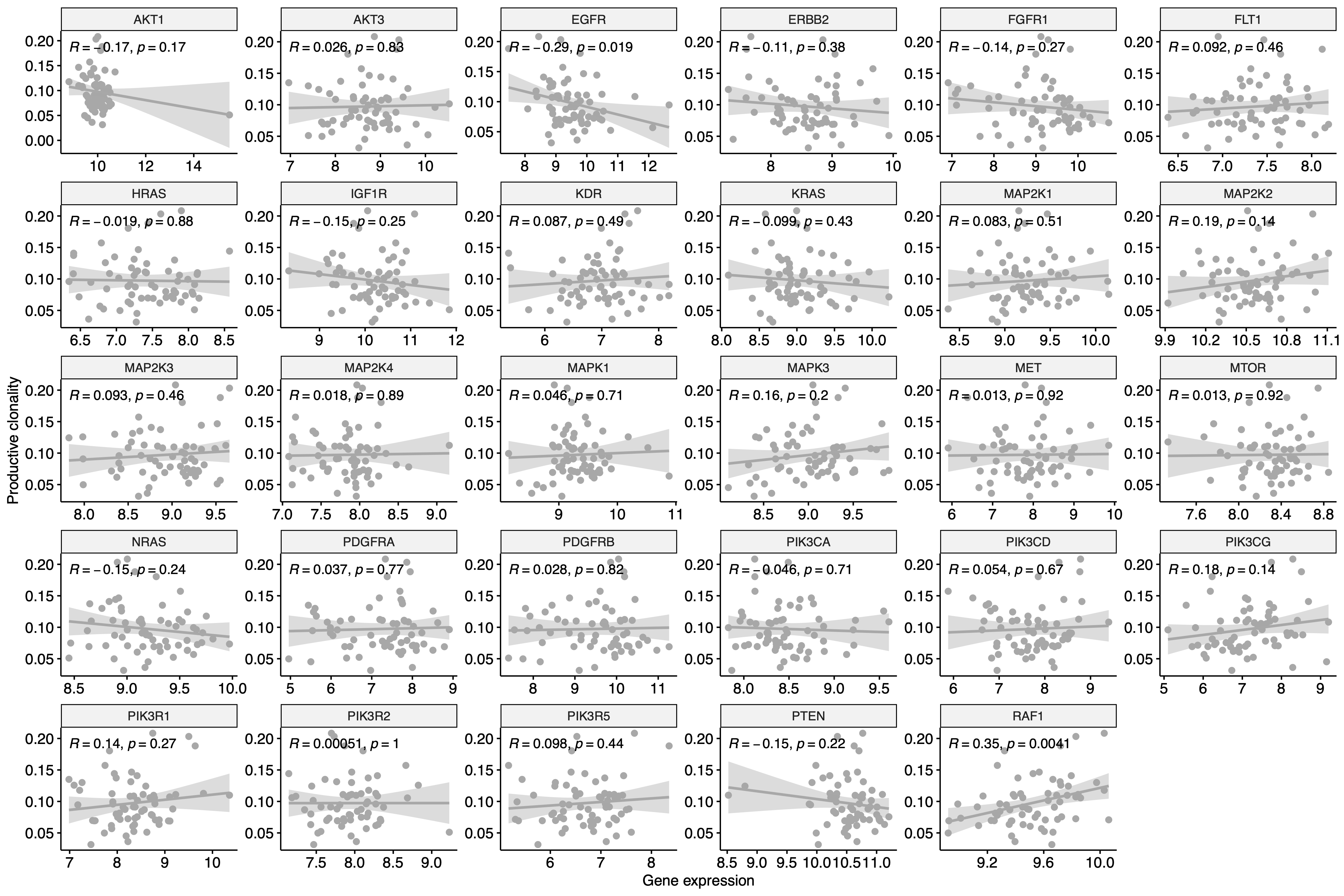


**Supplementary Figure 14.** Correlation between the expression of genes in the Ras/MAPK and PI3K/AKT pathway and TCR productive clonality. Only EGFR shows a borderline significant negative correlation.


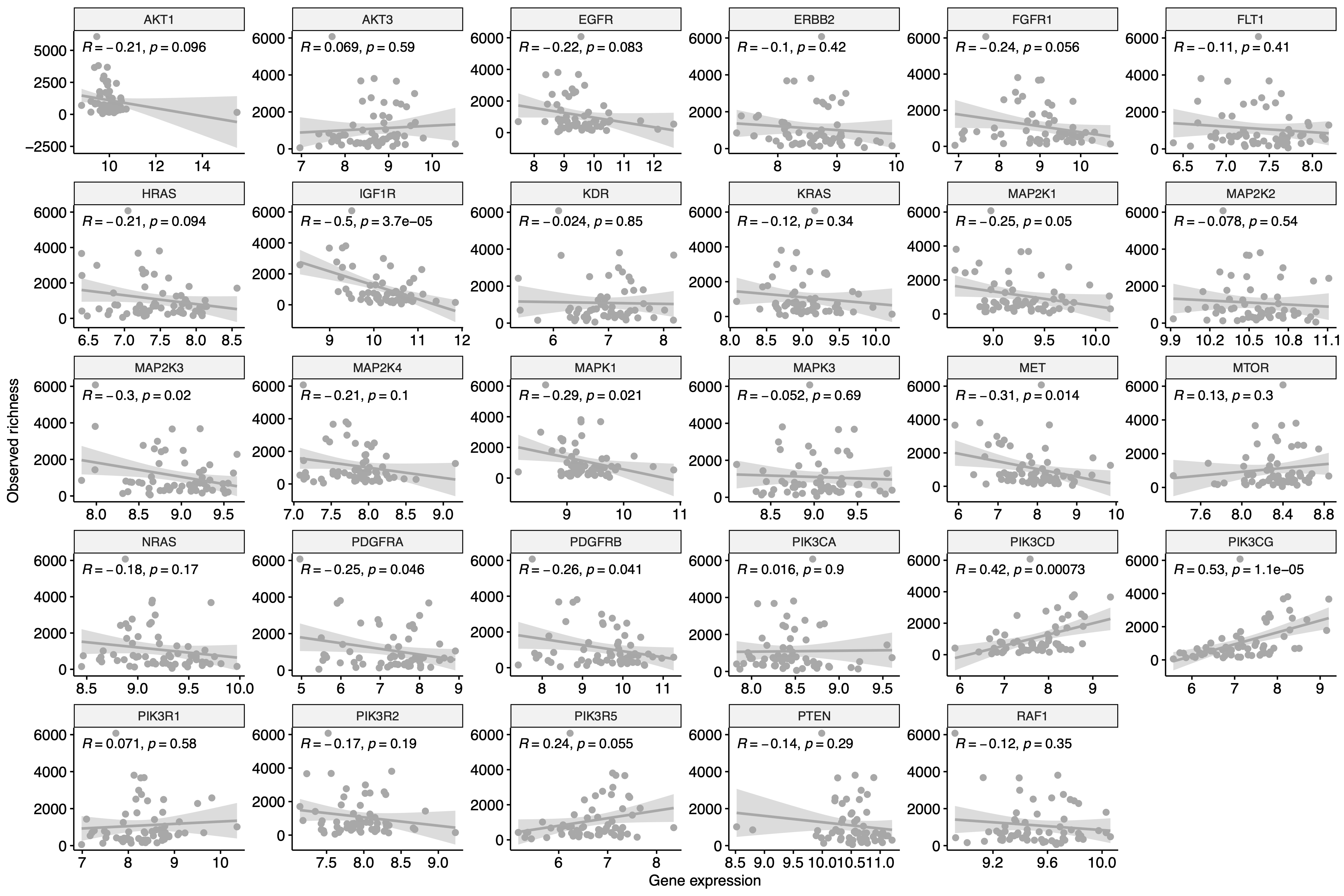


**Supplementary Figure 15.** Correlation between the expression of genes in the Ras/MAPK and PI3K/AKT pathway and the observed richness of the TCR repertoire.


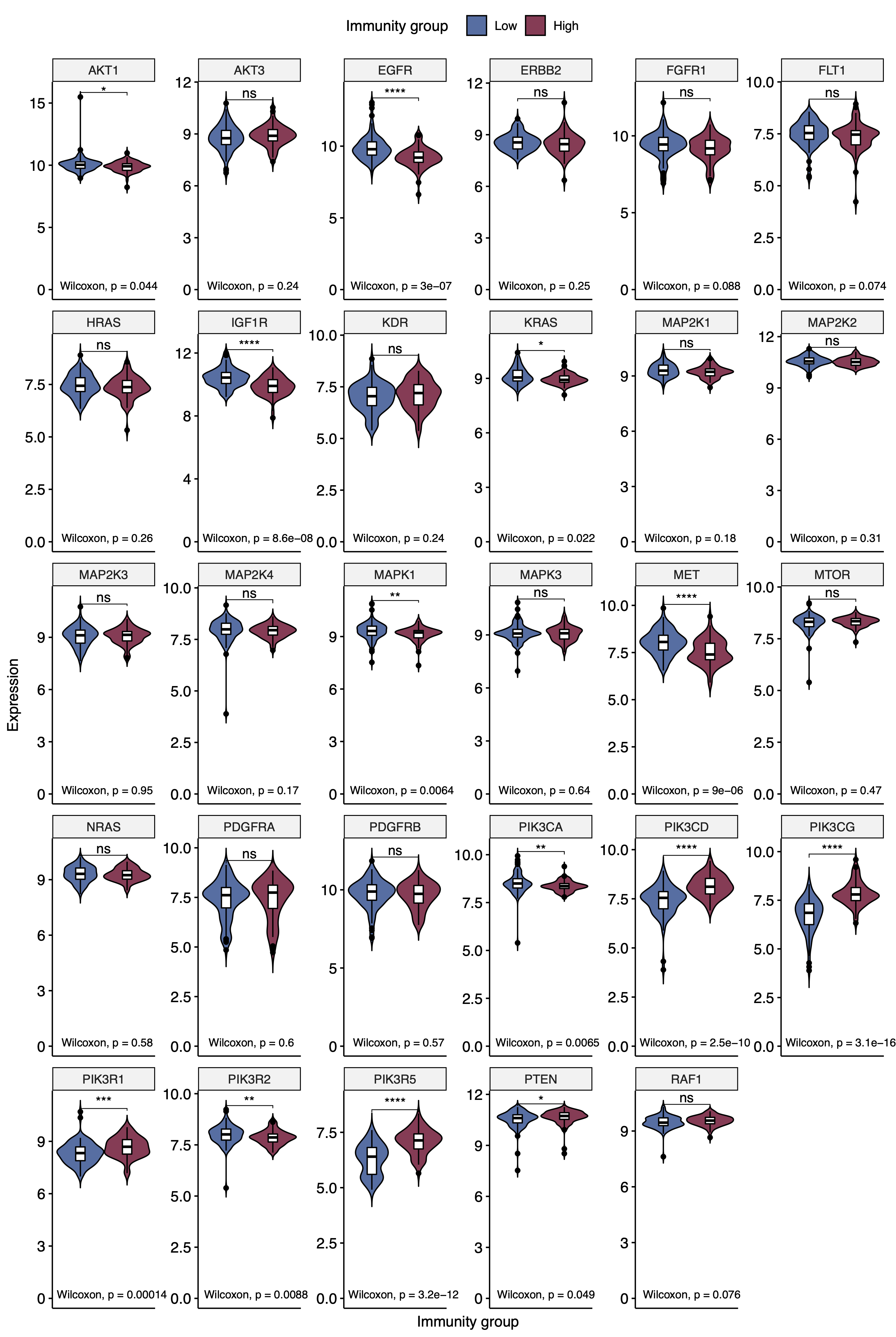


**Supplementary Figure 16.** Expression of 29 (out of 52) receptor tyrosine kinases and downstream genes in the MAPK/ERK and PI3K/AKT pathways was measurable using the Nanostring gene expression panel. There were significant differences in the level of expression for several of these genes between the two immunity classes, particularly for EGFR, IGF1R, KRAS, MAPK1, MET, PIK3CA, PIK3CD, PIK3CG, PIK3R1, PIK3R2, PIK3R5, PTEN. **** p<0.00001; *** p<0.0001; ** p<0.001; * p<0.05; ns – non-significant (p>0.05).


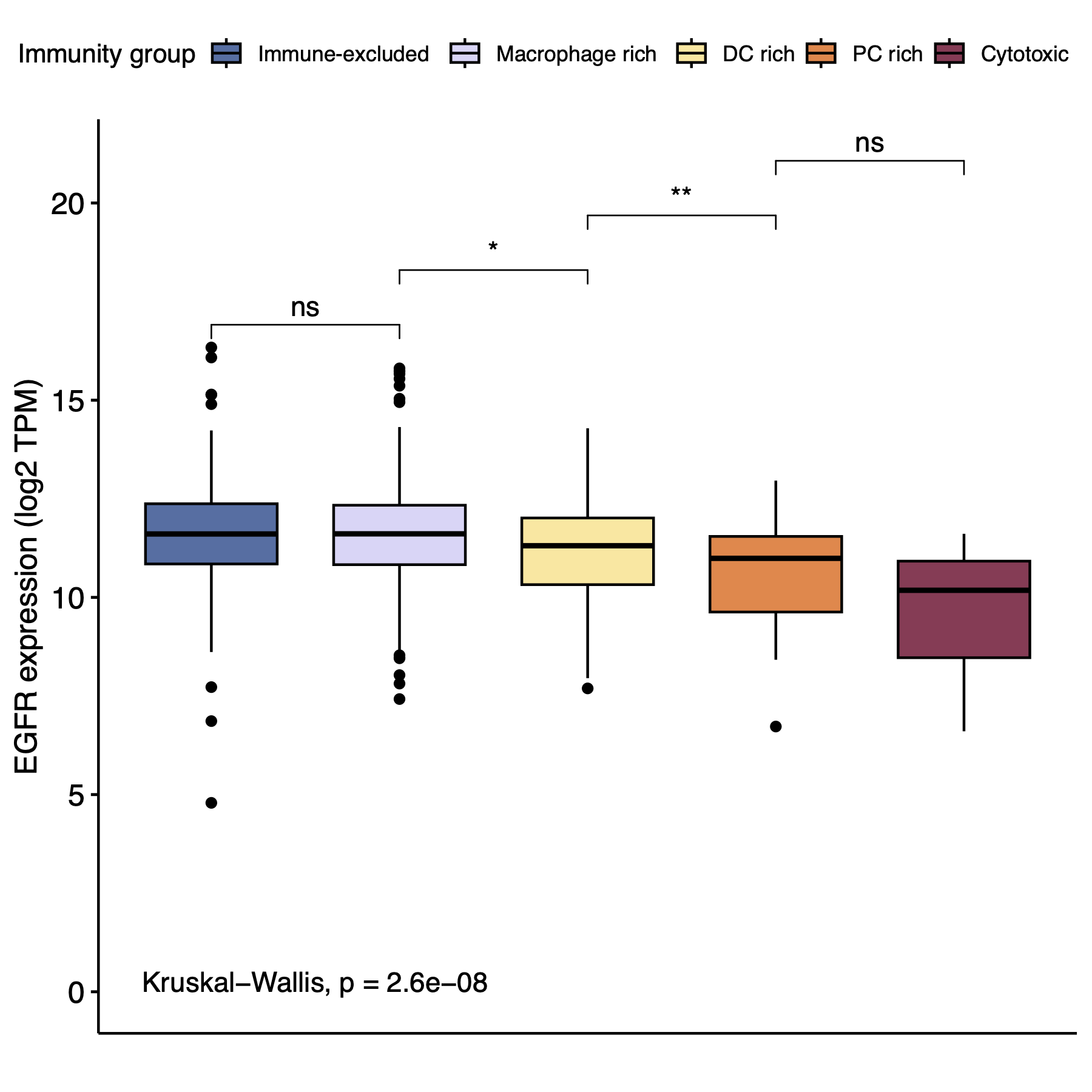


**Supplementary Figure 17. Validation of EGFR expression trends by immunity group in TCGA.** The expression of EGFR in tumours classed into the five immune categories is compared. ** p<0.001; * p<0.05; ns – non-significant (p>0.05).


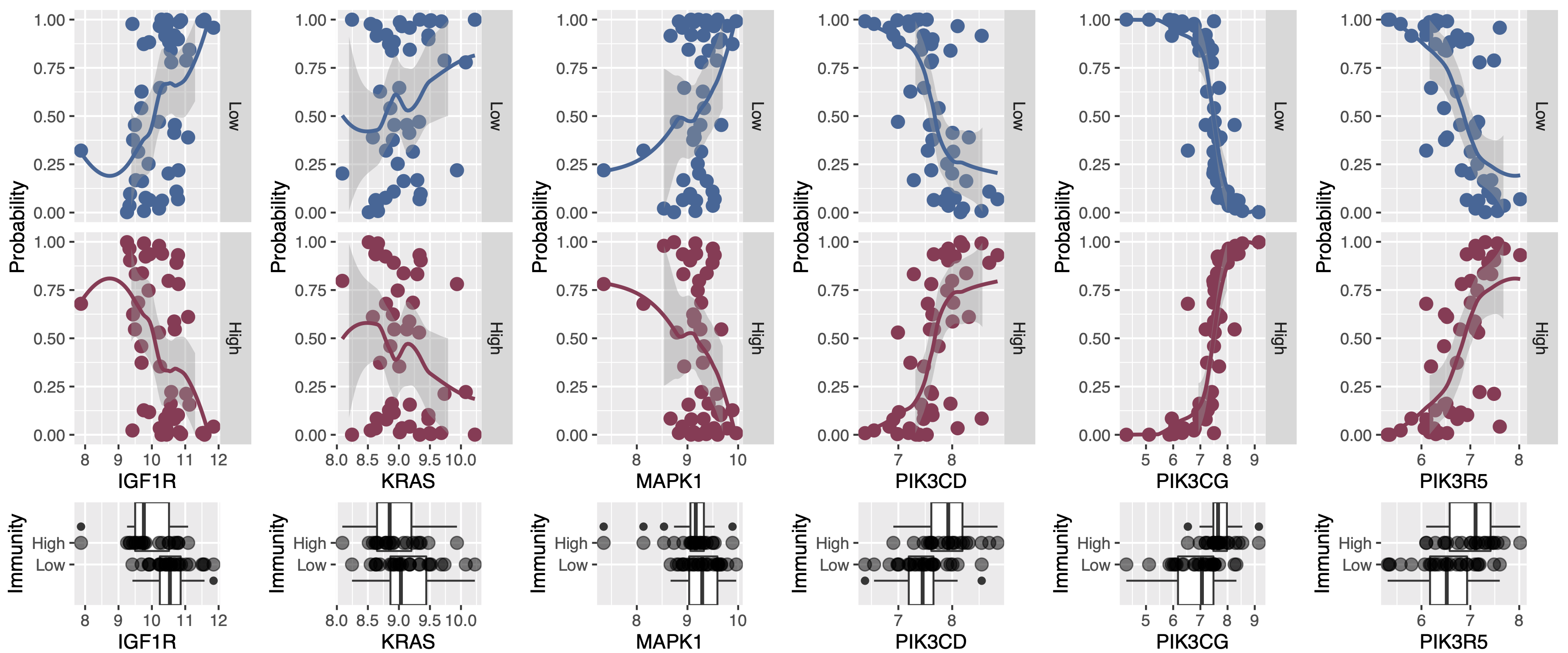


**Supplementary Figure 18. Modelling the two immunity classes (high/low) based on signalling of all measurable genes across tyrosine kinase pathways.** The genes included in the final model and their predictive power to distinguish between immunity classes are highlighted. Extreme values of the expression distribution are predictive of immune state in some cases.


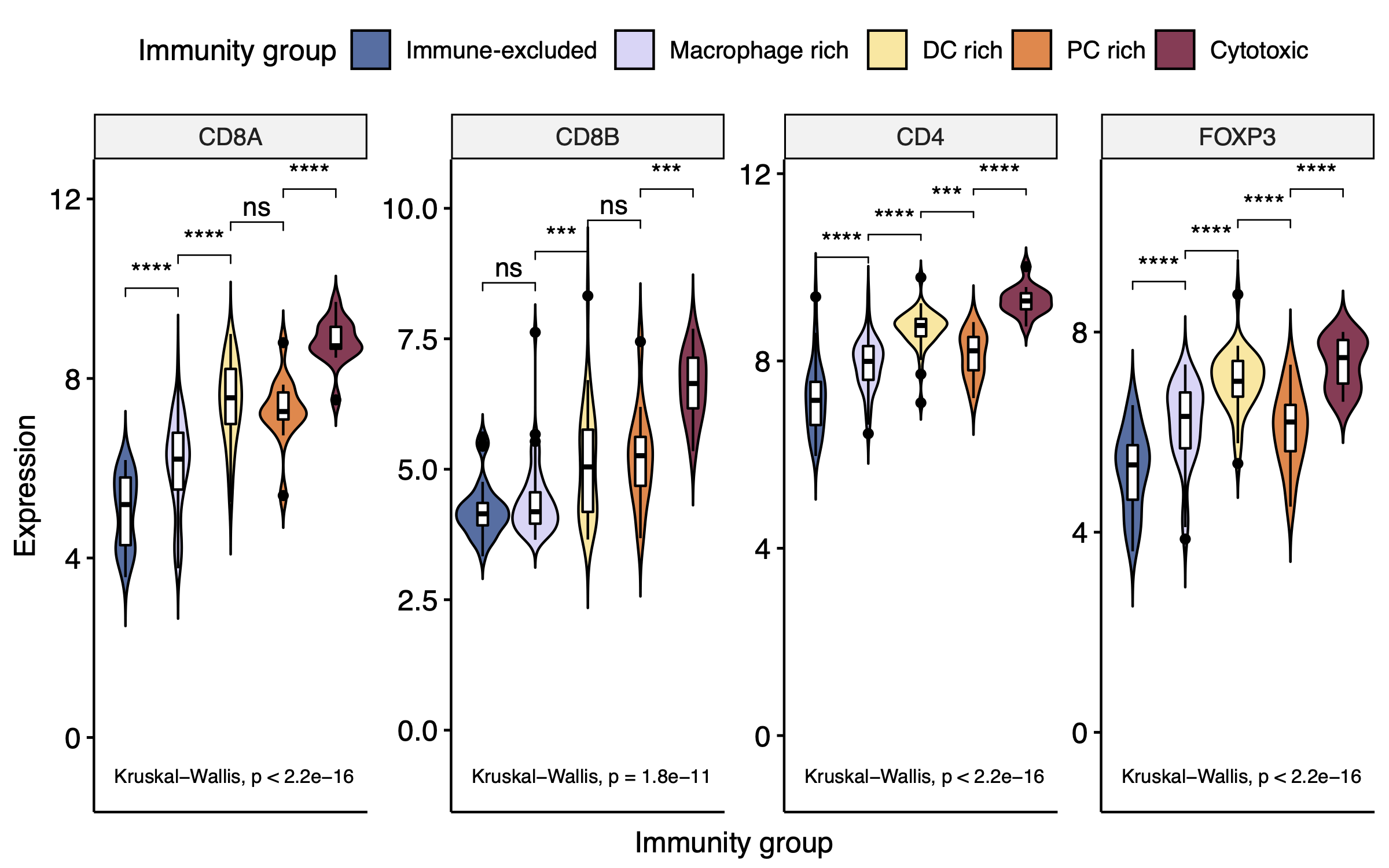


**Supplementary Figure 19. Expression of markers associated with high cytotoxicity across the immunity groups.** The expression of CD8A, CD8B, CD4 and FOXP3 genes is significantly elevated in the highly cytotoxic and exhausted groups, as expected. FOXP3, a marker generally associated with regulatory T cells, shows highest expression in the plasma cell enriched group. **** p<0.0001; *** p<0.001; ** p<0.01; * p<0.05; ns – non-significant (p>0.05).


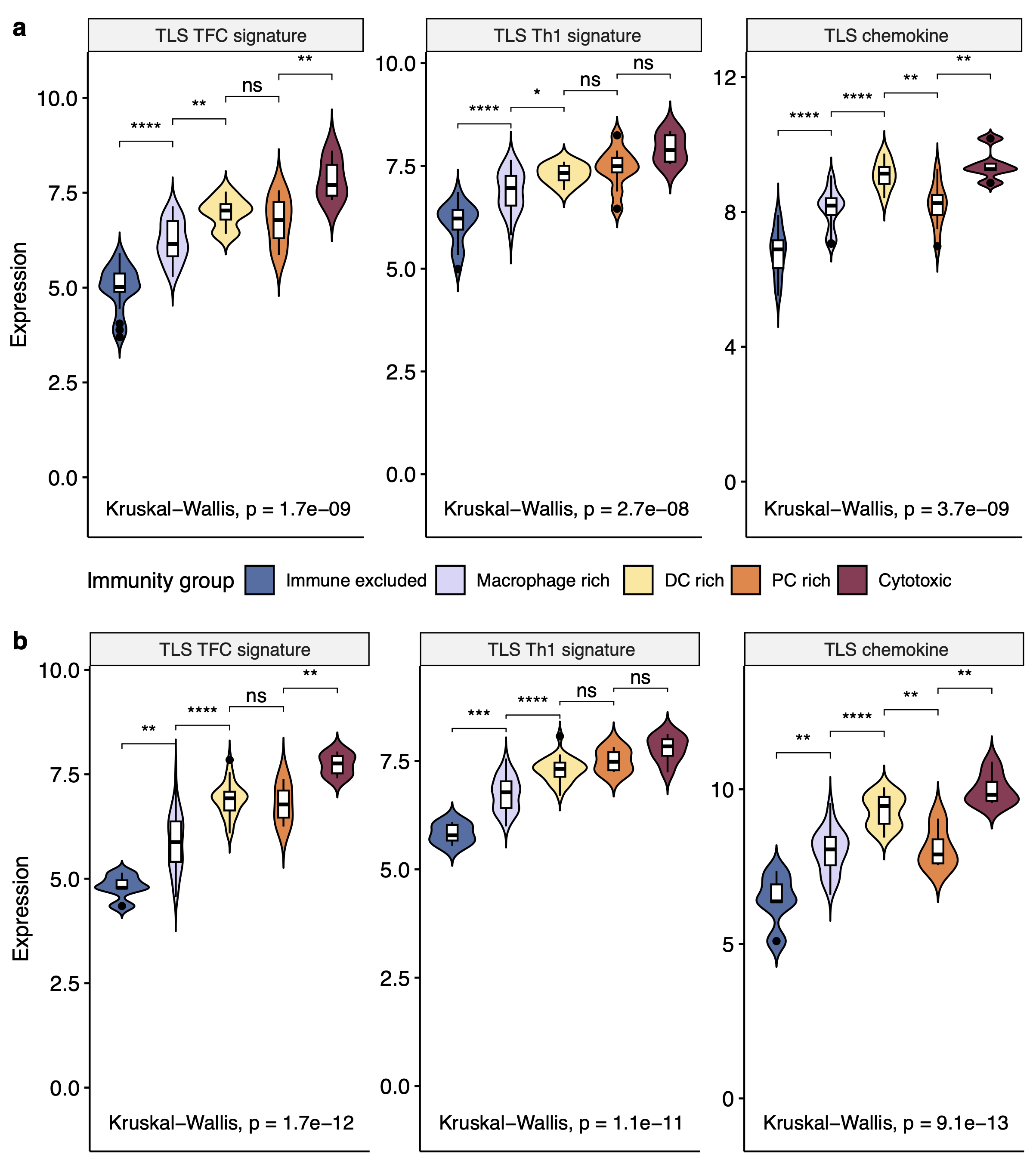


**Supplementary Figure 20. Expression of TLS-specific signature genes and chemokine signalling by HPV status.** The signatures are compared across immunity groups in (a) HPV positive cancers and (b) HPV negative cancers. Similar trends are observed in either cohort, and also similar to the amalgamated cohort. ****p<0.0001; ***p<0.001; **p<0.01; *p<0.05; ns – non-significant (p>0.05).


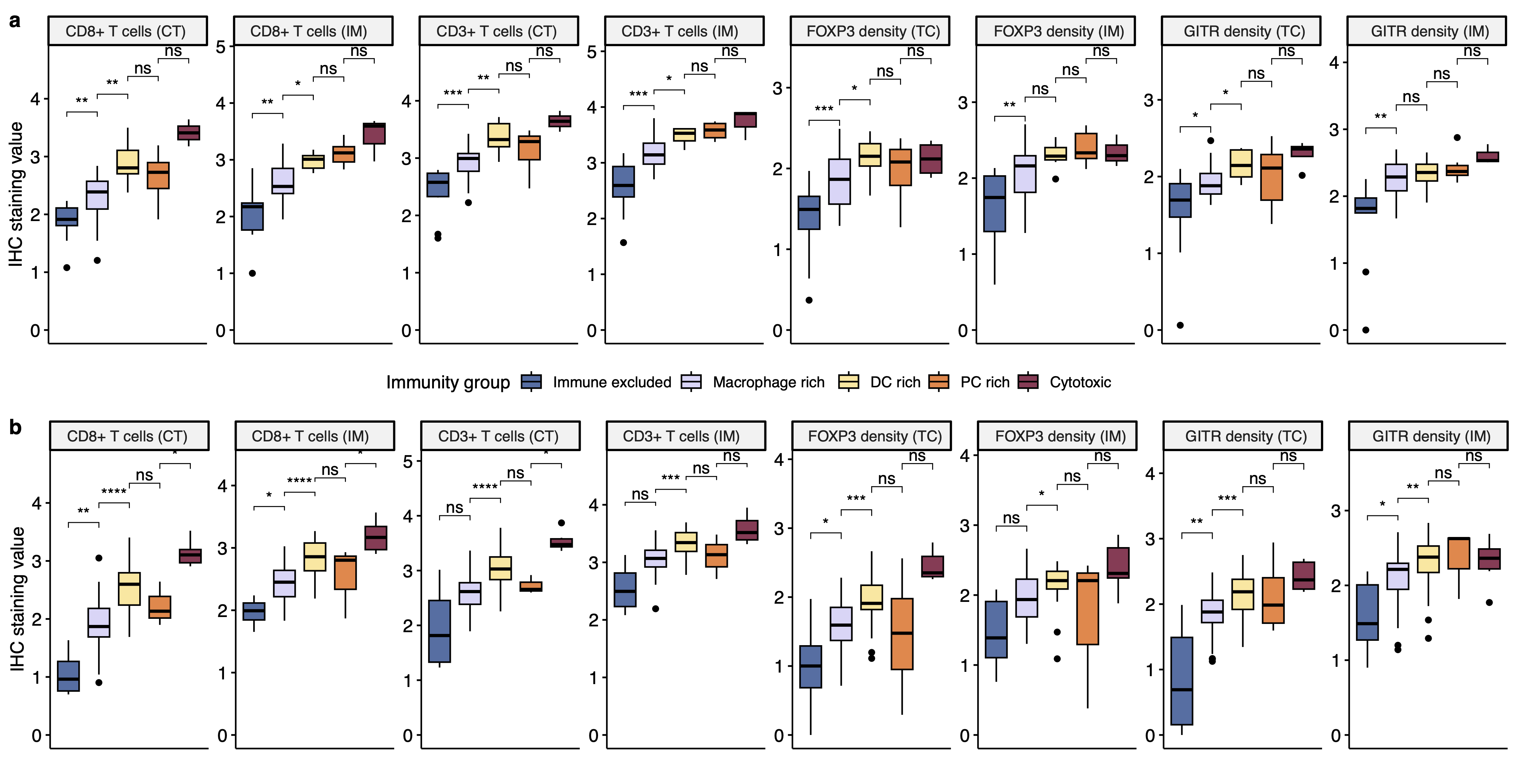


**Supplementary Figure 21. IHC protein staining for CD8+/CD3+ T cells and Treg markers (FOXP3, GITR) by HPV status.** Boxplots compare staining levels of relevant markers between independent samples across the five groups within(a) HPV positive cancers and (b) HPV negative cancers. TC – staining at the tumour centre; IM – staining at the immune margin. ****p<0.0001; ***p<0.001; **p<0.01; *p<0.05; ns – non-significant (p>0.05).


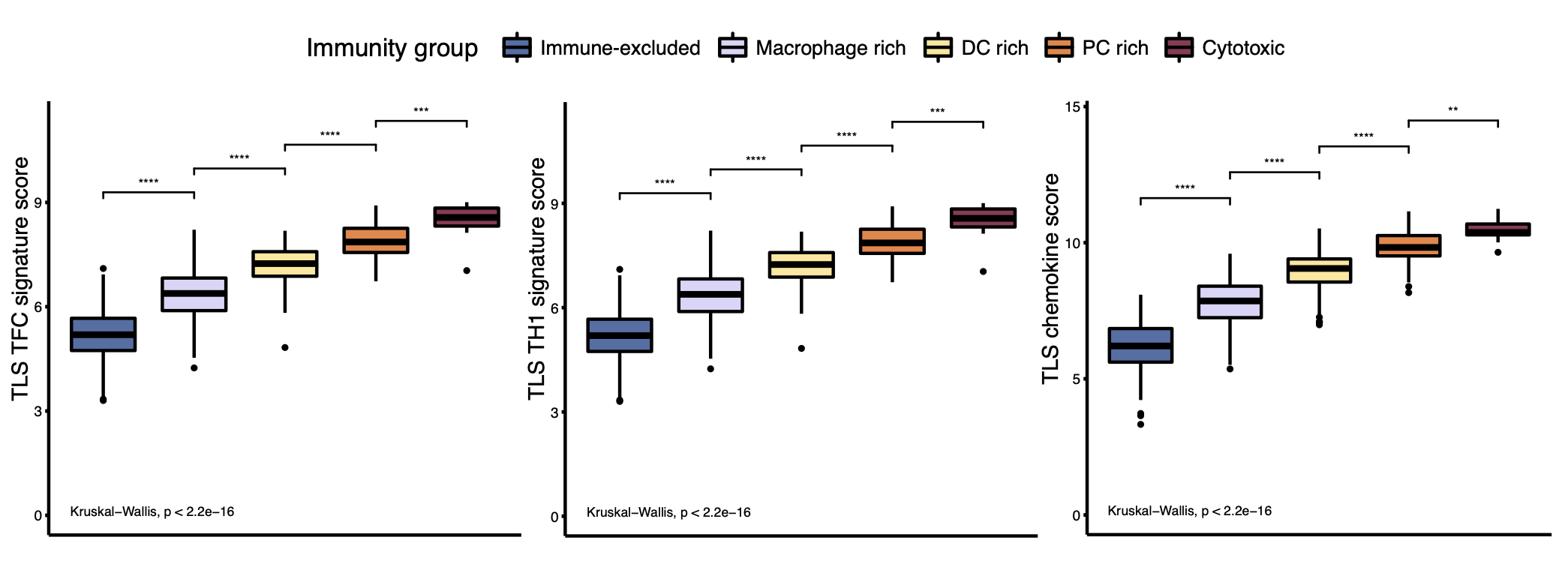


**Supplementary Figure 22. Validation of TLS signature trends in TCGA.** The TLS TFC, TH1 and chemokine scores increase significantly between low immunity and high immunity groups across 520 TCGA HNSCC samples. The cytotoxic group has the highest average of TLS signature scores. **** p<0.00001; *** p<0.0001; ** p<0.001.


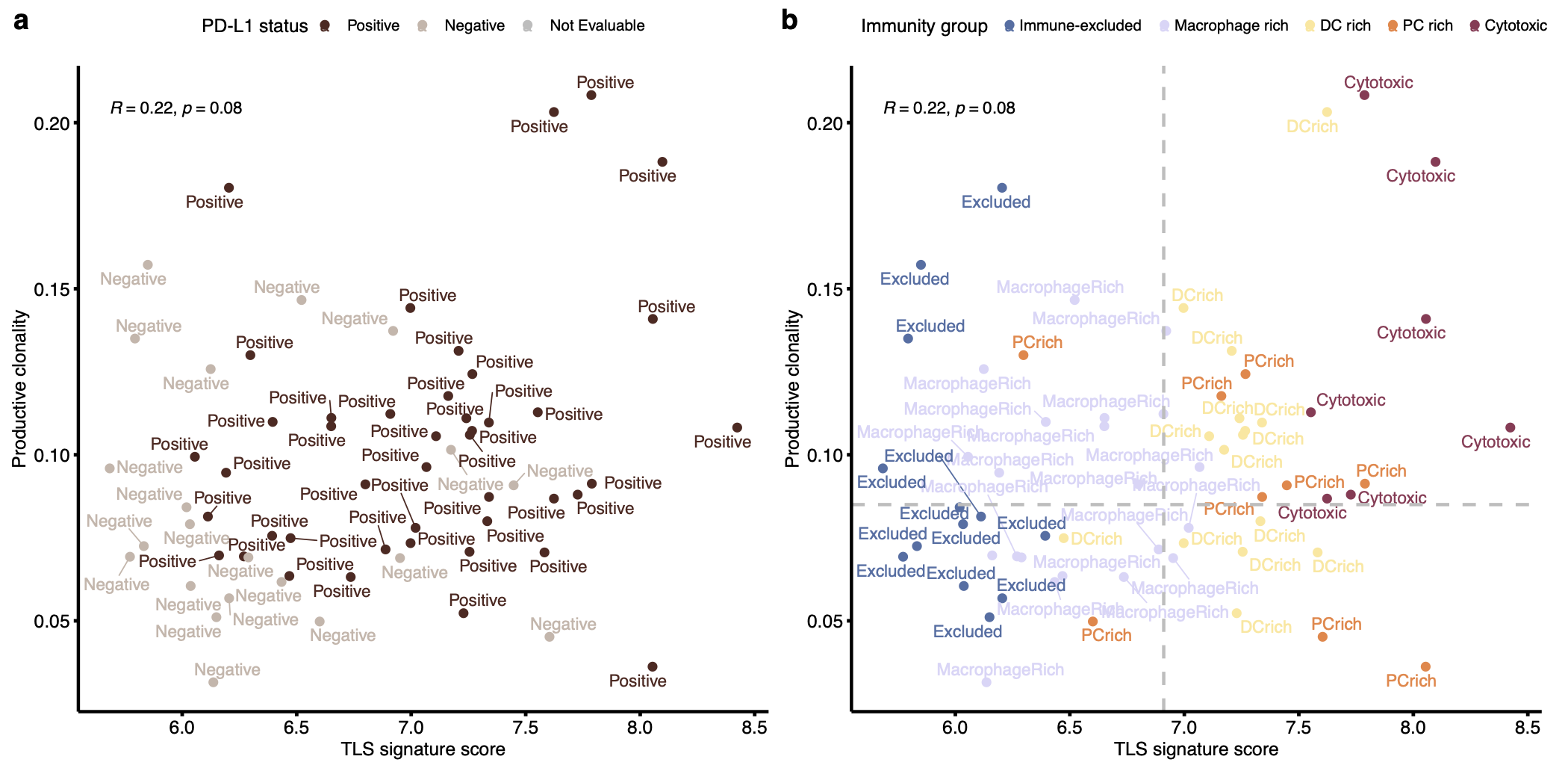


**Supplementary Figure 23.** The TLS signature score is weakly correlated with the productive clonality of the TCR repertoire. Data points are labelled according to (a) PD-L1 status by immunohistochemistry and (b) immunity group. Cytotoxic tumours present high productive clonality and high TLS signature scores most often, while immune-excluded and macrophage rich tumours tend to show lower clonality and lower TLS signature scores.
